# Network Pharmacology‐Based and Experimental Validation Elucidate the Target Mechanism of Vinorine in Ameliorating Secondary Brain Injury After Intracerebral Hemorrhage

**DOI:** 10.1111/cns.70609

**Published:** 2025-09-25

**Authors:** Jia‐Wei Wu, Yi‐Ting Zhou, Bing‐Xin Wang, Peng Wang, Xu‐Qi Zhang, Shi‐Qing Du, Xiao‐Jie Lu, Zeng‐Li Miao, Xu‐Dong Zhao

**Affiliations:** ^1^ Department of Neurosurgery, Wuxi No. 2 People's Hospital Afliated Wuxi Clinical College of Nantong University Wuxi Jiangsu China; ^2^ Department of Neurosurgery Jiangnan University Medical Center Wuxi Jiangsu China; ^3^ Wuxi Neurosurgical Institute Wuxi Jiangsu China; ^4^ Wuxi School of Medicine Jiangnan University Wuxi Jiangsu China; ^5^ Department of Intervention Therapy The Afliated Hospital of Jiangnan University Wuxi Jiangsu China; ^6^ Department of Neurosurgery, Medical School of Nantong University Nantong University Nantong Jiangsu China

**Keywords:** ICH, JAK–STAT pathway, microglia, molecular docking, network pharmacology, Vinorine

## Abstract

**Background:**

Intracerebral hemorrhage (ICH) is a severe stroke subtype associated with high mortality and long‐term disability, for which no effective treatment currently exists. Vinorine (Vin), a monoterpene indole alkaloid derived from *Rauvolfia reflexa*, has been traditionally used for age‐related neurological disorders, yet its therapeutic potential and mechanisms in ICH remain unclear.

**Methods:**

An ICH mouse model was established via intracranial collagenase injection. Vin was administered intraperitoneally at varying doses, and its effects on motor function, sensory deficits, and neural regeneration were evaluated. Network pharmacology was employed to predict potential targets and pathways, followed by validation through molecular docking, in vivo experiments, and in vitro assays.

**Results:**

Network pharmacology identified four core targets and 35 related pathways, with JAK2 as a central node. In vivo, Vin significantly improved motor deficits, reduced cerebral edema, preserved blood–brain barrier integrity, and promoted hematoma resolution. These effects were mediated through modulation of the CXCR2–JAK–STAT axis and suppression of JAK2 phosphorylation. In vitro, Vin inhibited JAK–STAT activation in microglia, downregulated MMP expression, and facilitated the transition from M1 to M2 phenotypic polarization. Additionally, Vin enhanced the expression of neuronal markers (NF200, PSD95, GAP43) and reduced neuronal apoptosis.

**Conclusion:**

Vin attenuates neurological deficits after ICH likely by inhibiting MMP expression in microglia via regulation of the CXCR2–JAK–STAT pathway. These findings highlight the therapeutic potential of Vin and provide mechanistic support for its further development as a treatment for ICH.

## Graphical abstract

This schematic shows Vinorine inhibits the activation of the CXCR2‐JAK–STAT pathway axis mediated by microglia after intracerebral hemorrhage by targeting the JAK2 protein, thereby reducing the release of metalloproteinases, protecting the blood–brain barrier function, and reducing neuronal apoptosis, indicating that it has a certain effect in alleviating secondary brain damage after intracerebral hemorrhage.

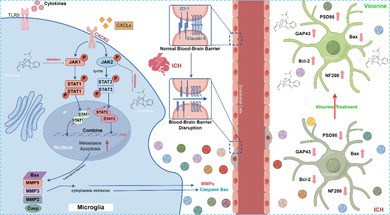

## 1 Introduction

Intracerebral hemorrhage (ICH) is a life‐threatening subtype of stroke, accounting for approximately 10%–5% of all stroke cases. It is characterized by high mortality and morbidity and currently lacks effective pharmacological treatments [1, 2]. Brain injury following ICH comprises both primary and secondary components. Primary brain injury results from the immediate mechanical compression caused by hemorrhage and hematoma expansion. Hematoma volume is a well‐established biomarker for ICH severity and is predictive of clinical outcomes [3, 4]. However, studies have shown that early hematoma evacuation does not significantly improve patient prognosis [4, 5], prompting a shift in research focus toward secondary brain injury. Secondary brain injury is a delayed pathological process triggered by the lysis of erythrocytes within the hematoma. The release of hemoglobin and iron exacerbates cerebral edema [6], neuroinflammation [7], cytotoxicity [8], and neuronal damage [9]. Despite extensive research efforts, there is currently no effective treatment to mitigate secondary brain injury following ICH. Natural products have long served as a valuable source of pharmacologically active compounds. In recent years, increasing attention has been directed toward plant‐derived agents for neurological protection. Compounds such as alkaloids [10], flavonoids [11], and terpenoids [12] have demonstrated neuroprotective effects and represent promising candidates for the development of novel therapeutics for ICH.

Monoterpene indole alkaloids are a class of natural alkaloids biosynthesized through the condensation of tryptophan and secologanin to form strictosidine, which then undergoes various modifications to yield structurally diverse derivatives [13]. The unique chemical structures and broad bioactivities of these alkaloids have attracted considerable scientific interest. In recent decades, increasing evidence has demonstrated that indole alkaloids possess diverse pharmacological activities, like anti‐inflammatory [14], antimicrobial [15], anticancer [16], and neuroprotective effects [17]. Vinorine (Vin), a representative monoterpene indole alkaloid, is predominantly derived from plants of the Apocynaceae family, particularly the *Alstonia* genus, which comprises approximately 60 species primarily distributed in tropical regions and is a major source of these alkaloids [13, 18]. Traditionally, species within the Apocynaceae family have been used in the treatment of age‐related neurological disorders. In Asia, Vin has been mainly isolated from the bark and leaves of *Rauvolfia reflexa* in India [17, 19] and *Alstonia scholaris* in China [20]. Notably, reserpine—a well‐known indole alkaloid with antihypertensive and centrally acting sedative properties—is also extracted from *Rauvolfia reflexa* and belongs to the same alkaloid group as Vin [19]. Although some studies have reported that Vin promotes axonal regeneration and facilitates motor and sensory recovery following sciatic nerve injury [17], its pharmacological effects in the context of ICH have not yet been explored.

The Janus kinase/signal transducer and activator of transcription (JAK–STAT) pathway is widely expressed in the central nervous system (CNS) and plays a critical role in regulating cell growth, differentiation, and inflammatory responses [21, 22]. Dysregulation of this pathway has been implicated in abnormal cell proliferation, neuroinflammation, neurodegeneration, and various CNS disorders [23]. In recent years, numerous studies have documented aberrant activation of the JAK–STAT pathway following ICH [24], which influences post‐ICH prognosis by modulating astrocyte proliferation [25] and contributing to neuroinflammation via microglial polarization [26]. Our previous findings also demonstrated that inhibition of the JAK–STAT pathway after ICH mitigates neuroinflammation and protects neurons from injury induced by pathological changes in the extracellular environment [27]. In the CNS, matrix metalloproteinases (MMPs) function as zinc‐dependent endopeptidases secreted predominantly by activated microglia and are essential for extracellular matrix (ECM) remodeling [28]. Under pathological conditions, excessive MMP expression contributes to tissue degradation and inflammation [29], adversely affecting neuronal survival. Moreover, MMPs are pivotal in the modulation of blood–brain barrier (BBB) integrity, and their expression is highly sensitive to neuroinflammatory stimuli [29, 30].

This study aimed to determine whether Vin enhances functional recovery following ICH and to elucidate its underlying mechanisms through cellular and animal models. Vin treatment distinctly attenuated neurological deficits, improved cognitive function, promoted hematoma clearance, and reduced neuronal apoptosis after ICH. In vitro, Vin preserved axonal protein expression and inhibited neuronal apoptosis. These neuroprotective effects were associated with its ability to modulate microglial activation and downregulate MMP expression, likely through inhibition of the JAK–STAT pathway.

## 2 Materials and Methods

### 2.1 Reagents and Chemicals

Vinorine (V962845, purity ≥ 98%) was obtained from McLean Chemical Co. Informasi terkait Vinorine terdapat pada (Table 1). Dimethyl sulfoxide (DMSO, HY‐Y0320), hemin (HY‐19424), CXCR2 inhibitor (CXCR2i, HY‐101022), CXCR2 agonist (CXCR2a, HY‐P4846), AG490 (HY‐12000), and Itacitinib (HY‐16997) were purchased from MedChemExpress (Shanghai, China). Collagenase VII (Batch No. C0773) was obtained from Sigma‐Aldrich (St. Louis, MO, USA). Primary antibodies against CXCR2 (DF7095), TLR9 (DF2970), GAPDH (AF7021), JAK1 (AF5012), phospho‐JAK1 (AF2012), JAK2 (AF6022), phospho‐JAK2 (AF3024), STAT1 (AF6300), phospho‐STAT1 (AF3300), STAT3 (AF6294), and phospho‐STAT3 (AF3293) were brought from Affinity Biosciences (Jiangsu, China). Secondary antibodies (goat anti‐mouse IgG and goat anti‐rabbit IgG) were procured from Cell Signaling Technology (Danvers, MA, USA). The BCA protein assay kit and ECL chemiluminescence detection reagent were procured from Biosharp (Anhui, China). DMEM high‐glucose medium (PM150210), fetal bovine serum (FBS, 164210), penicillin–streptomycin solution (100×, PB180120), and 0.25% trypsin–EDTA (PB180227; prepared in D‐Hank's) were obtained from Prosperity Life Sciences Co. Ltd. (Wuhan, China). Enhanced CCK‐8 reagent (C0043) was supplied by Beyotime Biotechnology (Shanghai, China).

**TABLE 1:** Basic information about Vinorine.

Formula	Molecular weight	GI absorption	BBB permeant	tPSA	Lipinski
C_21_H_22_O_2_N_2_	334.41 g/mol	High	Yes	41.09 Å^2^	Yes

### 2.2 Animal Model and Experiment Design

All procedures were granted by the Animal Ethics Committee of Jiangnan University (approval no. JN.No20240515c0160472 [054]) and followed ARRIVE guidelines for laboratory animal care. A total of 120 female C57BL/6J WT mice (6 weeks old, 19–21 g, SPF grade) were procured from Changzhou Cavens Laboratory Animal Co. and maintained in the Jiangnan University Animal Facility (License No. SYXK (SU) 2021‐0056) under standard conditions (20°C–25°C, 45%–55% humidity) with 1 week of acclimatization prior to experimentation.

The ICH model was established utilizing type VII collagenase [27]. Under isoflurane anesthesia (RWD Life Sciences, R510‐22), mice were secured in a stereotaxic frame, and 0.5 μL of type VII collagenase (0.075 U) was stereotactically delivered into the right caudate nucleus (1.0 mm anterior, 2.0 mm lateral, 3.5 mm deep relative to bregma). The needle was left in place for 10 min to minimize backflow. After sealing the cranial burr hole with bone wax, mice recovered on a 37.5°C heating pad. Mice that died spontaneously during induction or failed to develop contralateral hemiparesis within 24 h post‐injection were excluded from further analysis.

In the first experimental set, 100 mice were randomized into six groups (*n* = 14–16 per group): (1) sham‐operated, (2) ICH model, (3) Vin 7.5 mg/kg, (4) Vin 15 mg/kg, (5) Vin 30 mg/kg, and (6) edaravone 30 mg/kg. Except for the sham group, all mice received collagenase‐induced ICH. Dosages of Vin and edaravone were selected based on previous studies [17, 31]. Treatments were administered intraperitoneally beginning 24 h post‐ICH and repeated every 24 h thereafter. Short‐ and long‐term neurological function was examined on days 1, 3, 7, 14, and 35 post‐ICH. On day 3, mice were euthanized for brain tissue collection and hematoma analysis. In the second experimental set, 20 mice were randomized into three groups (*n* = 5–6 per group): (1) ICH model, (2) CXCR2 agonist (CXCR2a), and (3) CXCR2 inhibitor (CXCR2i). All mice received ICH induction. CXCR2a and CXCR2i were administered at doses referenced from prior studies [32, 33]. Mice were euthanized (Compliant with AVMA guidelines) 3 days post‐ICH for Western blot analysis of brain tissues.

### 2.3 Assessments of Neurological Function

Short‐term neurological function was tested utilizing the grid walking test and adhesion removal test at 1, 3, 7, and 14 days post‐ICH by three researchers who did not know the groupings, as previously described [27, 34]. In the grid walking test, mice were placed on an elevated wire grid and allowed to walk freely for 3 min. Normal mice maintain balance by gripping the grid, while ICH‐induced motor deficits result in paw slips through the grid gaps, referred to as “foot faults.” The number of foot faults and total steps were recorded for each limb. The foot fault index was calculated as: (number of contralateral sides—number of ipsilateral sides)/total number of steps. A score of 0 indicates symmetrical limb use, while a positive score reflects contralateral motor impairment. In healthy mice, the foot fault rate typically remains below ±5% [27]. In the adhesive removal test, small adhesive tape dots were alternately applied to each forepaw. Mice were gently restrained, and the times to first contact and complete removal of the adhesive were recorded. Each forepaw was tested three times, and mice were habituated to the cage for 2 min prior to testing. To minimize variability, animals were trained daily for three consecutive days before ICH induction to familiarize them with the procedure and exclude outliers.

Spatial learning and memory were evaluated utilizing the Morris water maze (MWM) by three researchers who did not know the groupings [35]. The test was implemented in a circular black pool (180 cm diameter, 50 cm height) filled with water at 22°C–24°C and divided into four quadrants. A 10 cm‐diameter transparent platform was submerged 2 cm below the surface in the target quadrant. The experiment comprised two phases: acquisition (place navigation) and retention (probe trial). Before training, mice were habituated by allowing free swimming without a platform for 1 min. During the training phase, mice were introduced facing the wall from different quadrants and given 90 s to locate the hidden platform. If unsuccessful, they were gently guided to the platform and allowed to remain there for 20 s. Trials were spaced at least 15 min apart. Escape latency and swim speed were tracked using an overhead video system. On day 35 post‐ICH, spatial memory was evaluated in a probe trial. The platform was removed, and mice were placed in the quadrant opposite to where the platform had been. They were allowed to swim freely for 60 s. Time spent in the target quadrant and the number of crossings over the previous platform location were recorded for analysis.

### 2.4 Brain Water Content (BWC) Measurement

Brain edema was quantitatively assessed using the wet/dry weight method at 1, 3, and 7 days following ICH induction, following established protocols [36]. Under deep isoflurane anesthesia, mice were euthanized, and brain regions—including ipsilateral and contralateral basal ganglia and cortex—were rapidly isolated and weighed to determine wet weight. Tissues were then dried at 100°C for 24 h to obtain dry weight. BWC was calculated utilizing the formula: (wet weight‐dry weight)/wet weight × 100%.

### 2.5 Evans Blue (EB) Extravasation and Fluorescence

BBB permeability was tested utilizing EB dye as described previously [37]. Mice received a 2% EB solution (5 mL/kg; Sigma‐Aldrich, E2129) via tail vein injection. After 2 h, animals were anesthetized and perfused with saline. Brains were harvested, and EB distribution in the right hemisphere was examined macroscopically. For fluorescence analysis, a separate cohort was perfused with saline followed by 4% paraformaldehyde (PFA). Brains were sectioned coronally at 16 μm and counterstained with DAPI. EB fluorescence was visualized utilizing a Zeiss Axio Imager2 fluorescence microscope (Germany).

### 2.6 Measurement of the Hemorrhage Volume

ICH hematoma volume was measured on day 3 post‐injury, as previously described [31]. Brains were removed and sectioned coronally into five evenly spaced slices (2 mm thick). Hemorrhagic (red/pink) areas were quantified by an investigator blinded to group assignments using ImageJ software (v1.5, NIH, USA). Hemorrhage volume was computed by summing the hemorrhagic areas across all slices and multiplying by the section thickness.

### 2.7 Histological Analysis and Nissl Staining

Freshly harvested organs were fixed in 4% PFA overnight at ambient temperature, paraffin‐embedded, and sectioned at 5 μm thickness. After deparaffinization and rehydration through graded ethanol, sections were stained with hematoxylin and eosin (H&E). Imaging was completed utilizing a Leica DM6 microscope (Germany). For Nissl staining, sections were incubated in 1% toluidine blue solution preheated to 50°C [38], followed by staining at 56°C for 20 min. Sections were then rinsed with double‐distilled water, differentiated with graded ethanol, and examined microscopically until clear neuronal structures and lesion sites were visible.

### 2.8 Data Mining for Network Pharmacology

**FIGURE 1:**
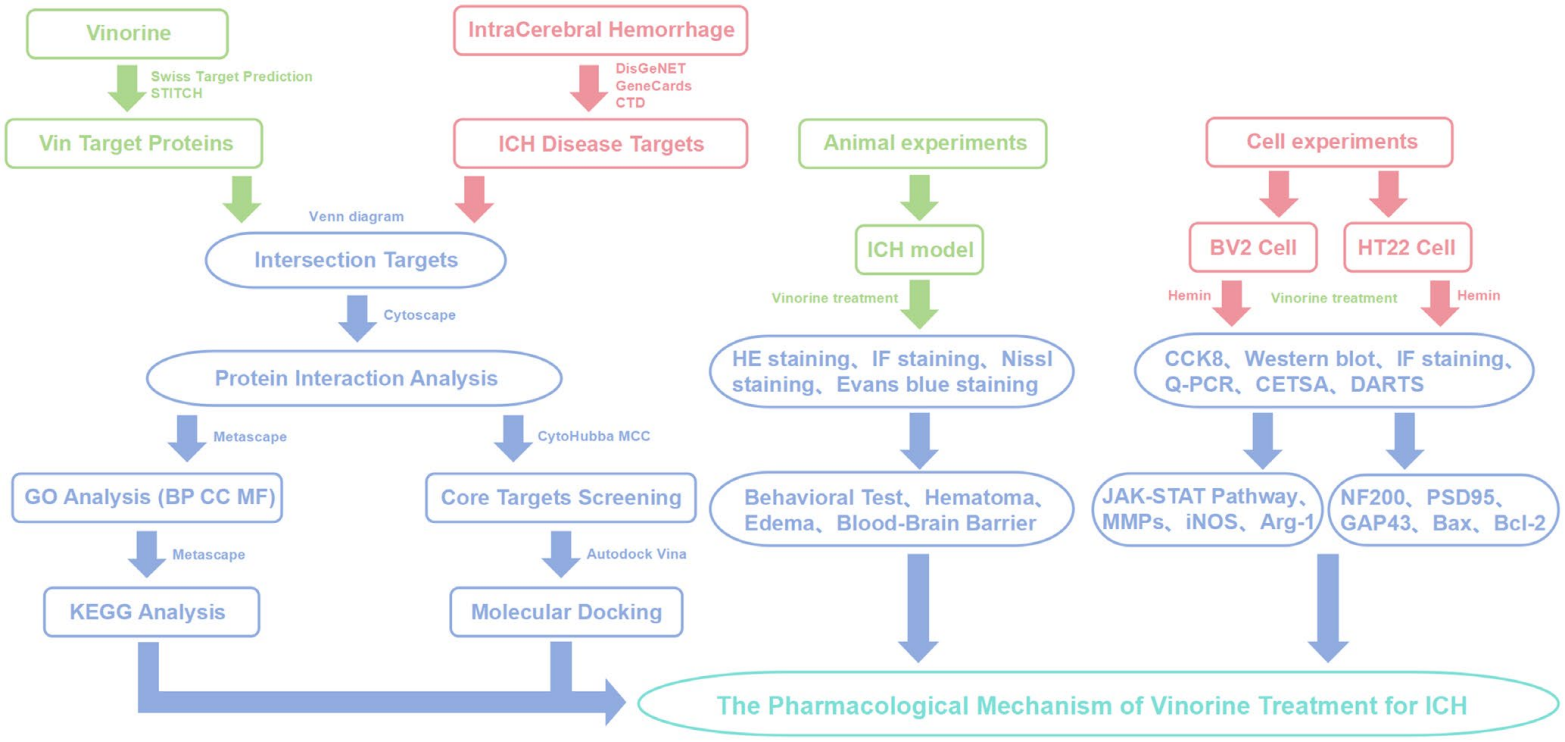
A schematic diagram illustrating the target mechanism of Vinorine treatment for ICH revealed by network pharmacology methods.

The network pharmacology analysis was conducted as previously described [27]. Potential targets of Vin were identified using the SwissTargetPrediction database (http://www.swisstargetprediction.ch/). ICH‐related genes were retrieved from the DisGeNET (https://www.disgenet.com/), GeneCards (https://www.genecards.org/), and Comparative Toxicogenomics Database (CTD; https://www.ctdbase.org/). The overlapping targets between Vin and ICH‐related targets were identified utilizing the Venny online tool (https://bioinfogp.cnb.csic.es/tools/venny/). Subsequently, the intersecting target genes were input into the STRING database (https://cn.string‐db.org/), and the results were imported into Cytoscape software (version 3.10.1) for visualization analysis. The parameters of each node in the network diagram were calculated to display molecular connections. The target genes were ranked based on their degree values (the top 20 target genes by degree value are listed in Table 2), and the network was analyzed visually using the CytoHubba MCC algorithm to identify core target genes. To explore the biological significance of the intersecting targets, GO analysis—including biological process (BP), cellular component (CC), and molecular function (MF) categories—and KEGG pathway enrichment analysis were implemented utilizing the Metascape platform (https://metascape.org/gp/index). A schematic of the web‐based pharmacological analysis workflow is presented in Figure 1.

**TABLE 2:** Information about the top 20 PPI networks.

Name	Average shortest path length	Betweenness centrality	Closeness centrality	Degree
CASP3	1.69565	0.21225	0.58974	40
MMP9	1.8913	0.0915	0.52874	34
PARP1	1.91304	0.08407	0.52273	24
TLR9	2.1087	0.03944	0.47423	24
CTSB	2.13043	0.09165	0.46939	24
MAOB	1.97826	0.0669	0.50549	22
JAK2	2.19565	0.02858	0.45545	22
MAOA	2.13043	0.04166	0.46939	20
PIK3CA	2.15217	0.02968	0.46465	20
SLC6A3	2.3913	0.03806	0.41818	18
MAPK14	2.08696	0.0802	0.47917	18
JAK1	2.26087	0.0061	0.44231	18
IDO1	2.13043	0.04933	0.46939	18
SLC6A4	2.3913	0.04189	0.41818	16
PGR	2.08696	0.04222	0.47917	16
ACHE	2.17391	0.03012	0.46	14
MME	2.21739	0.02446	0.45098	14
DRD2	2.63043	0.04793	0.38017	14
CTSK	2.23913	0.02099	0.4466	14
JAK3	2.34783	0.01067	0.42593	14

### 2.9 Molecular Docking and Molecular Dynamics (MD) Simulation

Molecular docking was completed following established procedures [39]. The crystal structures of JAK2, TLR9, Caspase 3, and MMP9 were retrieved from the Protein Data Bank (https://www.pdbus.org). Target proteins were prepared using AutoDock Tools (v1.5.7) by removing water molecules and adding hydrogen atoms, followed by conversion to .pdbqt format. The chemical structure of Vin was obtained from PubChem (https://pubchem.ncbi.nlm.nih.gov), energy‐minimized utilizing Chem3D, and converted to .sdf format. Vin was further processed in AutoDock Tools 1.5.7 by assigning atomic charges, setting all flexible bonds as rotatable, and exporting the ligand in .pdbqt format. Grid box parameters (center, size, and dimensions) were defined based on the predicted binding site of Vin to each target protein. Molecular docking was carried out using AutoDock Vina, and binding conformations were visualized using PyMOL (v4.6.0).

MD simulations were conducted in Discovery Studio 2019 (DS) to evaluate the dynamic behavior of the ligand‐protein complexes [40]. The simulation workflow consisted of the following steps: (1) Protein preparation: Protein‐ligand complexes were imported into DS, and the “Prepare Protein” protocol was applied to add hydrogen atoms, correct bond geometries, and repair missing residues; (2) System solvation: The CHARMM36 force field was used to solvate the complex by surrounding it with water molecules, establishing a realistic simulation environment; (3) Simulation setup: The “Standard Dynamics Cascade” protocol was employed with parameters set for a 100‐ns production run. The solvated system was designated as the active environment for simulation execution; (4) Post‐simulation analysis: Upon completion, trajectory data were analyzed for hydrogen bond dynamics, ligand‐protein interactions, and overall system stability. Root mean square deviation (RMSD) and root mean square fluctuation (RMSF) values were calculated to assess the structural consistency and flexibility of the complexes.

### 2.10 Real‐Time Quantitative PCR (RT‐qPCR)

Total RNA isolation from perihematomal brain tissue was done utilizing Trizol reagent (R401, Vazyme, Nanjing, China), and its purity was tested via NanoDrop spectrophotometry (Thermo Scientific, Rockford, IL, USA) [41]. cDNA synthesis was implemented employing a 5× All‐in‐One RT premix kit (R333‐01, Vazyme). qPCR was carried out with AceQ SYBR Green Master Mix (Q111‐02, Vazyme) on an Applied Biosystems 7500 system. All samples were run in triplicate. Gene expression was normalized to β‐actin and analyzed with the help of the 2^−ΔΔCt^ method. Primer sequences are provided in Table 3.

**TABLE 3:** Specific primers used for quantitative real‐time PCR.

Gene	Forward (5′–3′)	Reverse (5′–3′)	Number of bases
Mouse‐CXCL1	TCCAGAGCTTGAAGGTGTTGCC	AACCAAGGGAGCTTCAGGGTCA	22/22
Mouse‐CXCL3	TGAGACCATCCAGAGCTTGACG	CCTTGGGGGTTGAGGCAAACTT	22/22
Mouse‐ITGB1	CTCCAGAAGGTGGCTTTGATGC	GTGAAACCCAGCATCCGTGGAA	22/22
Mouse‐β‐Actin	CATTGCTGACAGGATGCAGAAGG	TGCTGGAAGGTGGACAGTGAGG	23/22
Mouse‐MMP2	CAAGGATGGACTCCTGGCACAT	TACTCGCCATCAGCGTTCCCAT	22/22
Mouse‐MMP3	CTCTGGAACCTGAGACATCACC	AGGAGTCCTGAGAGATTTGCG	22/21
Mouse‐MMP9	GCTGACTACGATAAGGACGGCA	TAGTGGTGCAGGCAGAGTAGGA	22/22
Mouse‐GAPDH	AGGTCGGTGTGAACGGATTTG	GGGGTCGTTGATGGCAACA	21/19

### 2.11 Cell Culture

The mouse hippocampal neuronal cell line HT22 (CL‐0697) and mouse microglial cell line BV2 (CL‐0493) were provided by Punosai Biotech (Wuhan, China). Cells were cultured in DMEM high‐glucose medium enriched with 10% heat‐inactivated FBS and 1% penicillin–streptomycin. Cultures were maintained at 37°C in a humidified incubator with 5% CO_2_. Vin and hemin stock solutions were dissolved in DMSO and diluted in culture medium to final concentrations, ensuring DMSO did not exceed 0.1% (v/v). Prior to treatment, cells were serum‐starved for 12 h in DMEM. For in vitro modeling of ICH, cells were exposed to 50 μM Hemin for 12 h, following established protocols [42].

### 2.12 Western Blot Analysis

Western blot was performed as described previously [43]. Briefly, perihematomal brain tissue (approximately 50 mg, 2 mm surrounding the hematoma) was collected on day 3 post‐ICH. Tissues were lysed in pre‐chilled RIPA buffer containing PMSF, protease inhibitors, and phosphatase inhibitors. Equal amounts of protein were separated by SDS‐PAGE and transferred to PVDF membranes (Millipore, Burlington, MA, USA). Membranes were blocked with 5% skim milk for 1–2 h at ambient temperature, followed by overnight incubation at 4°C with primary antibodies against JAK1 (1:1000), phospho‐JAK1 (1:1000), JAK2 (1:1000), phospho‐JAK2 (1:1000), MMP9 (1:1000), cleaved Caspase‐3 (1:1000), and GAPDH (1:5000). Subsequently, membranes were incubated with HRP‐conjugated secondary antibodies (1:3000) for 2 h at ambient temperature. Signal detection was implemented utilizing an ECL kit, and band intensities were quantified with ImageJ.

### 2.13 Immunofluorescence Staining

For tissue immunofluorescence, paraffin‐embedded brain tissue sections were deparaffinized, rehydrated, and subjected to antigen retrieval as previously described [44]. After blocking with 10% BSA at room temperature, sections were incubated overnight at 4°C with primary antibodies (1:200). Following washes, fluorescent secondary antibodies were applied for 1.5 h at ambient temperature. DAPI was adopted for nuclear counterstaining (15 min). Imaging was done with a Zeiss fluorescence microscope.

For cellular immunofluorescence [45], fixed cells (4% paraformaldehyde, 20 min) were permeabilized with 0.5% Triton X‐100 for 10 min, then blocked with 10% BSA for 1 h. Cells were incubated with primary antibodies (1:200, overnight at 4°C), followed by Alexa Fluor 488 or 594‐conjugated secondary antibodies (1:200; ab150080, ab150077; Abcam, USA) for 1 h in the dark. Nuclei were labeled with DAPI (15 min), and images were acquired utilizing a Zeiss fluorescence microscope under appropriate settings.

### 2.14 Cellular Thermal Shift Assay (CETSA)

CETSA was performed based on a previously established protocol [46]. BV2 cell lysates (100 μL) were treated with 25 μM Vin and subjected to a temperature gradient (50°C–71°C) in 3‐min increments. After heating, samples were centrifuged at 20,000 g for 10 min at 4°C. Supernatants were collected, combined with 5× loading buffer, and analyzed by 10% SDS‐PAGE followed by Western blotting.

### 2.15 Drug Affinity‐Responsive Target Stability (DARTS) Assay

DARTS was performed as previously described [47]. BV2 cell lysates were incubated with varying concentrations of Vin for 1 h at ambient temperature. Pronase E (5 μg/mL; HY‐114158, MedChemExpress, USA) was then added and allowed to digest for 45 min. The reaction was terminated by adding a loading buffer, and protein samples were subsequently analyzed via Western blot.

### 2.16 Statistical Analyses

All experiments were performed using randomized and blinded protocols. Each experiment was independently repeated at least four times, with a minimum of four biological replicates per group for both in vitro and in vivo studies. Statistical analyses were conducted using GraphPad Prism 10.0.2 (GraphPad Software Inc., La Jolla, CA, USA). Data are presented as mean ± standard deviation (SD). Before further statistical testing, data distribution was evaluated for normality. Upon confirmation of normal distribution, a two‐way analysis of variance (ANOVA) followed by Tukey's post hoc test was applied to assess group differences. Statistical significance was defined as follow: *, *p* < 0.05; **, *p* < 0.01; ***, *p* < 0.001; # or ****, *p* < 0.0001. No exclusion criteria were applied, and no outlier data were removed from the analysis.

## 3 Results

### 3.1 Vin Promotes Haematoma Resorption and Improves Neurological Deficits After ICH

**FIGURE 2:**
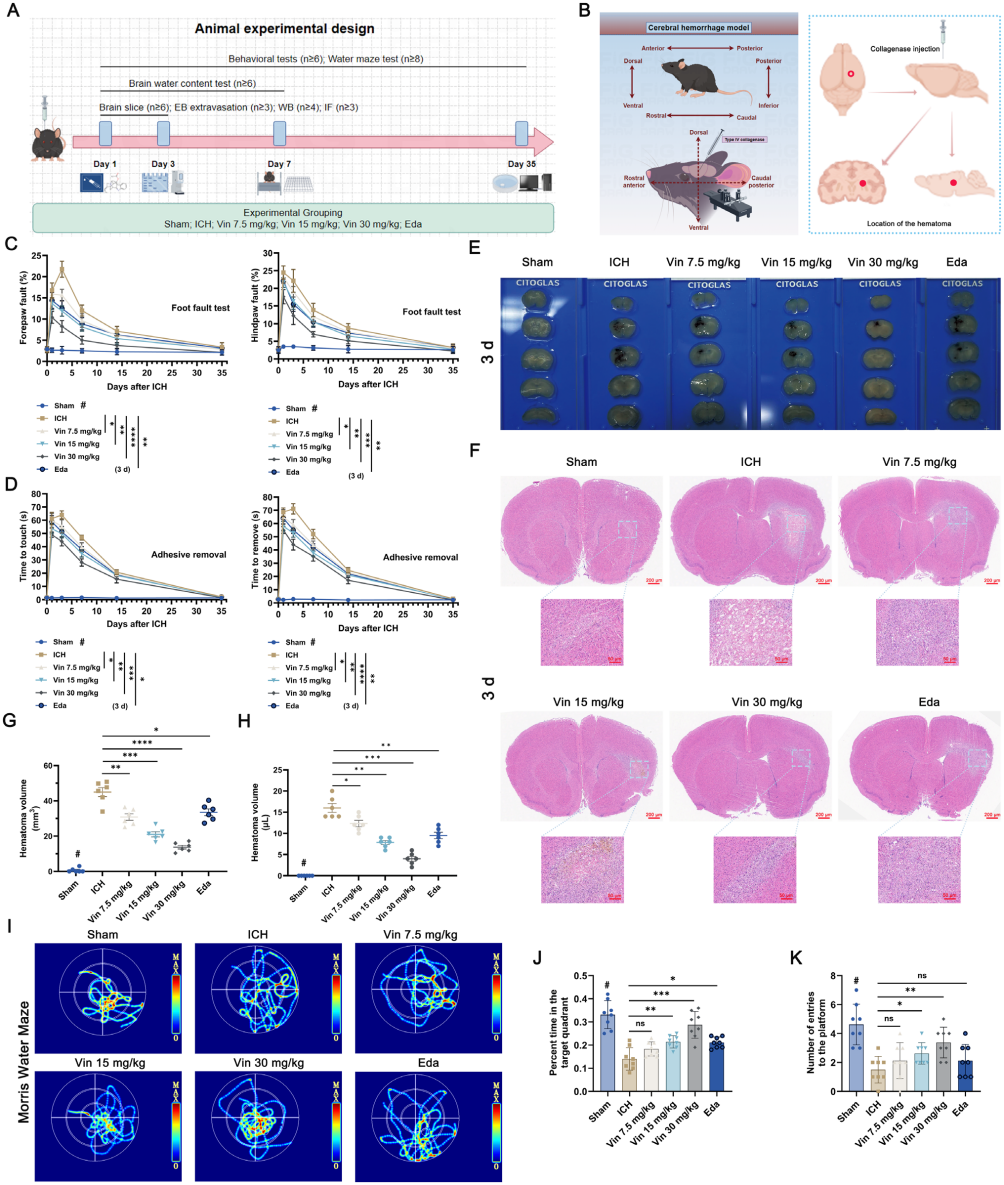
In vivo experiments investigating the therapeutic effects of Vinorine on ICH. (A) Animal experiment design and grouping diagram. (B) ICH model and hematoma location diagram. (C) Grid walking test: Foot error rate grid detection of the contralateral forelimb and hindlimb (*n* = 6). (D) Adhesion removal test: Adhesion removal time detection of the contralateral forelimb and hindlimb (*n* = 6). (E) Observation of haematoma size in brain tissue sections on the third day after ICH. (F) Representative images of hematoxylin and eosin (H&E) staining of brain sections. Scale bar: 50 μm. (G) Haematoma volume (*n* = 6). (H) Quantitative measurement of hemorrhage volume (*n* = 6). (I) Representative swimming path diagrams for each group in the water maze experiment (*n* = 8). The starting point is located in the second quadrant. (J) Time spent in the target quadrant by each group during the MWM test (*n* = 8). (K) Number of times each group of mice entered the platform during the MWM test (*n* = 8). All data were expressed as mean ± standard deviation (SD). Statistical significance was determined by two‐way analysis of variance (ANOVA) and Tukey's multiple comparisons test, *p < 0.05, **p < 0.01, ***p < 0.001 and # or ****p < 0.0001 VS ICH group, *n* ≥ 6. ns, not significant.

The overall experimental workflow and schematic diagram of the ICH model are shown in Figure 2A,B. To preliminarily assess Vin's safety, we evaluated its effects on major organs. H&E staining of the liver, kidneys, and other tissues revealed no notable histological changes across treatment groups (Figure S1A), and serum biochemical analyses showed no significant alterations in AST, ALT, UA, CREA, or LDH levels (Figure S1B), suggesting that Vin does not induce systemic toxicity. To evaluate the therapeutic effects of Vin on ICH, neurological function was assessed using the grid‐walking test, adhesive removal test, and MWM. Grid‐walking and adhesive removal were conducted on days 1, 3, 7, 14, and 35 post‐ICH. As depicted in Figure 2C, the foot fault rate was notably elevated following ICH intervention from day 1 to 14 (*p* < 0.001). Notably, Vin treatment evidently reduced foot faults at day 3 post‐ICH, particularly at 15 and 30 mg/kg doses (*p* < 0.05), indicating a dose‐dependent improvement. By day 35, differences between groups were no longer statistically significant (*p* > 0.05). In the adhesive removal test, ICH mice showed significantly prolonged contact and removal times on day 3 compared to sham controls (*p* < 0.001). Vin‐treated mice demonstrated significantly improved sensorimotor recovery, as indicated by shorter contact/removal times relative to the ICH group (Figure 2D), which indicated that Vin treatment effectively promoted the recovery of sensorimotor function.

To assess Vin's effect on hematoma clearance, H&E staining and coronal brain sections were analyzed on day 3 post‐ICH (Figure 2E,F). Hematoma formation was clearly evident in the ICH model group, confirming successful model establishment (Figure 2E). Edaravone significantly reduced hematoma volume compared to the ICH group (35.4 ± 5.36 mm^3^ vs. 45.3 ± 8.65 mm^3^, *p* < 0.05). Vin at 15 and 30 mg/kg further reduced hematoma volume to 21.36 ± 4.26 mm^3^ and 14.4 ± 3.28 mm^3^, respectively (*p* < 0.01 vs. ICH; Figure 2G). Intracerebral hemorrhage volume was also significantly decreased in the Vin 15 and 30 mg/kg groups (8.16 ± 1.68 μL and 4.31 ± 2.18 μL, respectively) compared to the ICH group (16.94 ± 3.28 μL, *p* < 0.05; Figure 2H). No significant differences were witnessed between the Vin 7.5 mg/kg and Edaravone groups. Histological analysis further revealed that neurons in the sham group were morphologically intact and regularly arranged. In contrast, neurons in the ICH group showed cytoplasmic loosening and nuclear condensation. Vin treatment reduced these pathological changes in a dose‐dependent fashion (Figure 2F).

Finally, long‐term cognitive function was assessed utilizing the MWM test on day 35 post‐ICH. No differences in swimming speed were observed among groups. Representative swim trajectories are illustrated in Figure 2I. In the spatial navigation trial, mice in the ICH group spent significantly less time in the target quadrant than those in the sham, Vin 15 mg/kg, and Vin 30 mg/kg groups (*p* < 0.05; Figure 2J). Platform‐crossing frequency was also reduced following ICH intervention (*p* < 0.01) and significantly improved in the 15 and 30 mg/kg Vin‐treated groups (*p* < 0.05; Figure 2K). No significant differences were noted among the ICH, Vin 7.5 mg/kg, and Edaravone groups (*p* > 0.05). Conclusively, Vin treatment promotes neurological recovery in ICH mice.

### 3.2 Vin Significantly Reduces Cerebral Oedema, Improves BBB Integrity, and Protects Neuronal Structure and Viability After ICH

**FIGURE 3:**
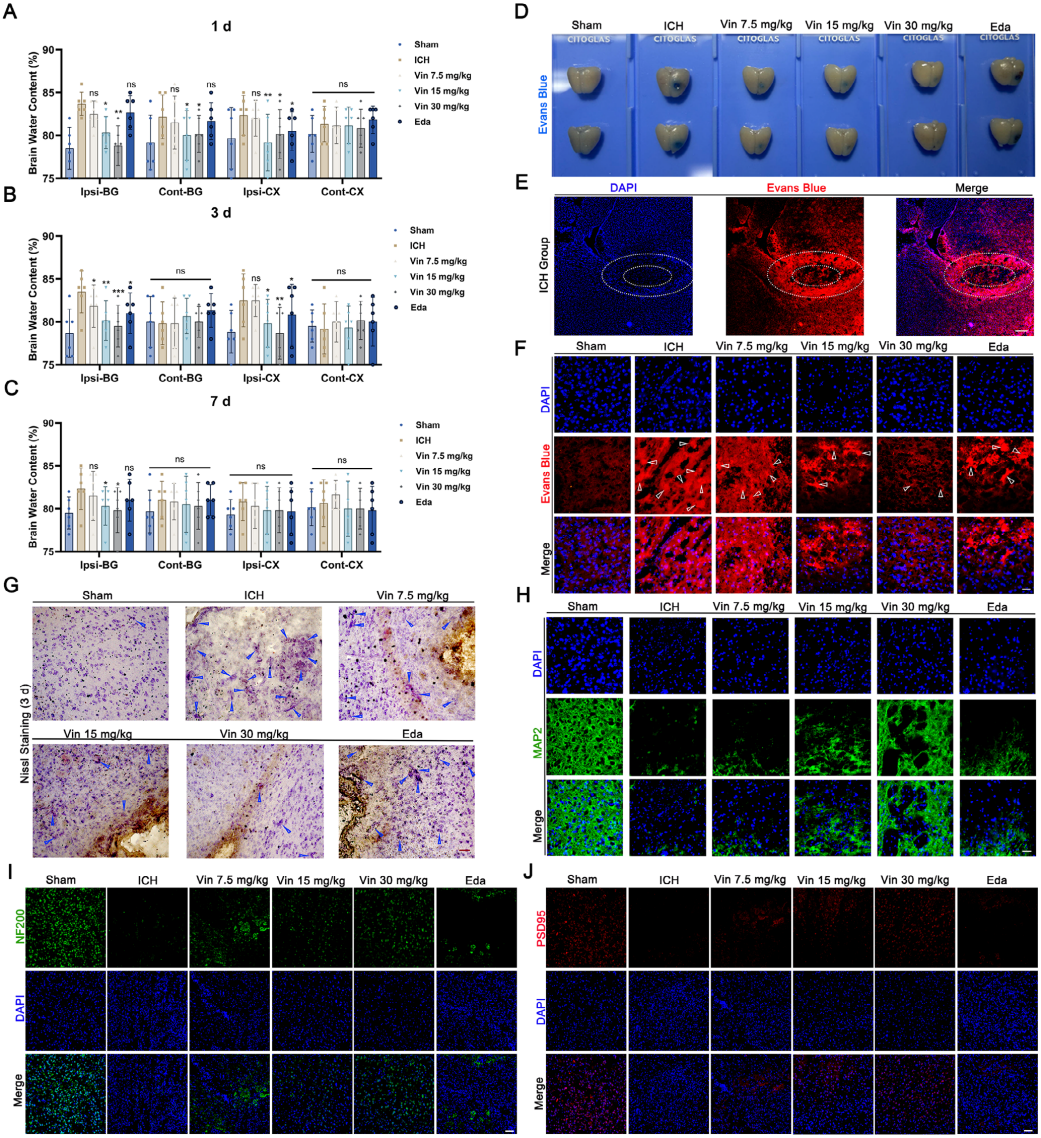
Effects of Vinorine on brain oedema, blood–brain barrier, and neurons following ICH. (A) Comparison of oedema severity in different brain regions of mice from each group 1 day after ICH (*n* = 6). (B) Comparison of oedema severity in different brain regions of mice from each group 3 days after ICH (*n* = 6). (C) Comparison of oedema severity in different brain regions of mice from each group 7 days after ICH (*n* = 6). (D) Comparison of Evans blue dye area in brain tissue after Evans blue tail vein injection (*n* = 6). (E, F) Evans blue fluorescence images. EB leakage occurs around the haematoma after ICH, with white arrows indicating EB dye extravasation. Scale bar = 50 μm. (G) Representative Nissl staining images of brain tissue from mice in each group 3 days after ICH. Scale bar = 50 μm. (H) Immunofluorescence staining of MAP2 (green) in brain tissue sections from each group of mice 3 days after ICH. Scale bar = 50 μm. (I, J) Immunofluorescence staining of NF200 (green) and PSD95 (red) in brain tissue sections from each group of mice 3 days after ICH. Scale bar = 50 μm. All data were expressed as mean ± standard deviation (SD). Statistical significance was determined by two‐way analysis of variance (ANOVA) and Tukey's multiple comparisons test, *p < 0.05, **p < 0.01, ***p < 0.001 VS ICH group, *n* ≥ 6. ns, not significant.

Cerebral edema was quantified by BWC analysis on days 1, 3, and 7 post‐ICH. Compared to the sham group, the ICH group exhibited a notable elevation in BWC in the ipsilateral basal ganglia and cortex on days 1 and 3, with a partial reduction observed in the ipsilateral cortex by day 7 (all *p* < 0.05; Figure 3A–C). Among brain regions assessed, the ipsilateral basal ganglia exhibited the most pronounced edema, and Vin treatment markedly reduced BWC at this site—particularly on day 3 (*p* < 0.05; Figure 3B). No significant changes were observed in the contralateral basal ganglia and cortex on days 3 and 7, although mild edema was detected in the contralateral basal ganglia on day 1 (*p* > 0.05; Figure 3B,C). Based on the temporal progression of cerebral edema and behavioral deficits, day 3 post‐ICH was selected as the principal time point for subsequent analyses. To evaluate BBB integrity, EB leakage assays were performed. On day 3 after ICH, mice exhibited distinctly increased EB extravasation in the ipsilateral (right) hemisphere (*p* < 0.05; Figure 3D and Figure S2A,B). Vin treatment significantly attenuated this leakage in a dose‐dependent manner. Correspondingly, EB fluorescence imaging revealed extensive leakage in the ICH group (white arrows), which was markedly reduced by Vin treatment (Figure 3E,F).

To further investigate neuronal injury and structural preservation, Nissl staining and immunofluorescence for MAP2 were conducted. Nissl staining revealed marked neuronal atrophy, membrane disruption, and cytoplasmic dissolution in the perihematomal region of the ICH group (indicated by blue arrows), consistent with significant neuronal damage. Vin treatment, particularly at 30 mg/kg, improved neuronal morphology and reduced tissue pathology (Figure 3G). Furthermore, immunofluorescence staining for MAP2, NF200, and PSD95 revealed extensive neurofilament fragmentation, shortening, and reduced axonal protein expression in the perihematomal region of the ICH group. Vin treatment preserved neurofilament structure in a dose‐dependent manner, suggesting enhanced neuronal survival and structural maintenance in the face of hematoma‐derived cytotoxicity (Figure 3H–J). The above results suggest that Vin treatment can effectively promote neuronal neurofilament and axonal protein survival after ICH and contribute to nerve repair around hematoma after ICH.

### 3.3 Network Pharmacological Correlation Analysis of Vin in the Treatment of ICH

**FIGURE 4:**
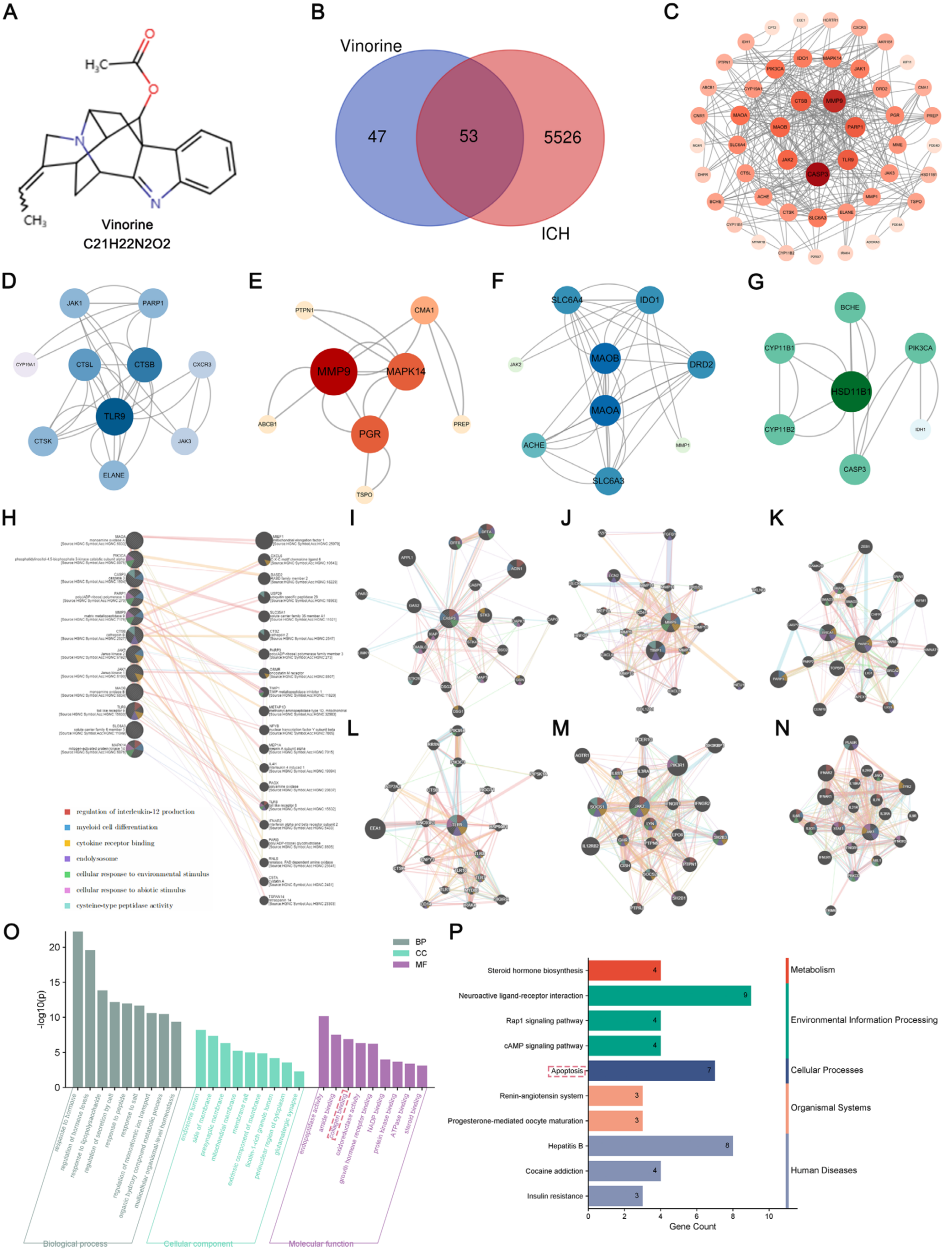
Network pharmacology analysis of key targets and mechanism pathways of Vinorine treatment for ICH. (A) Schematic diagram of the Vinorine chemical molecule. (B) Venn diagram of Vinorine and ICH targets. (C) Protein–protein interaction network of overlapping targets. (D–G) Subnetworks of protein–protein interactions with high connectivity were calculated using CytoHubba_MCC, with color intensity corresponding to weighted scores. (H) GenMANIA analysis of functional associations with the top twelve targets, with functional annotations of target proteins located in the bottom left corner. (I–N) Representative GenMANIA images for Cas3, MMP9, PARP1, TLR9, JAK2, and JAK1. (O) GO enrichment analysis of the intersecting targets, including biological processes, cellular components, and molecular functions. These enriched entries highlight key biological processes, cellular components, and molecular functions that may be influenced by Vinorine. (P) KEGG enrichment analysis plot of the intersecting targets, where the length of each bar represents the number of genes and indicates the enrichment score and significance level; longer bars indicate a higher number of genes and greater enrichment.

To investigate the potential molecular mechanisms and therapeutic targets of Vin in ICH, a comprehensive network pharmacology analysis was performed. The chemical structure of Vin is depicted in Figure 4A, and detailed pharmacological information is provided in Table 1. A total of 5579 ICH‐related genes were retrieved from the DisGeNET, GeneCards, and CTD databases. SwissTargetPrediction was utilized to identify 100 Vin‐related targets based on pharmacokinetic and structural similarity criteria. After deduplication and intersection, 53 common targets were validated as potential therapeutic targets of Vin for ICH (Figure 4B). The overlapping targets were input into the STRING database to generate a protein–protein interaction (PPI) network comprising 102 nodes and 1276 edges. The PPI network was visualized and analyzed in Cytoscape software, where node size and color intensity represent degree value, reflecting connectivity and relative importance (Figure 4C). The top 20 targets ranked by degree centrality are listed in Table 2. Further analysis using the CytoHubba plug‐in (MCC algorithm) identified four core hub genes that may serve as key mediators of Vin's therapeutic effects in ICH (Table 3). Module analysis using the MCODE algorithm revealed highly interconnected clusters involving these targets (Figure 4D–G). Among them, TLR9 and JAK2 emerged as top‐ranking nodes with strong network centrality. To explore functional associations, an extended gene interaction network was constructed using GeneMANIA. This analysis revealed 12 functionally related genes interconnected through 428 interactions (Figure 4H). Functional enrichment analysis indicated involvement in multiple biological processes, including protein kinase activation, regulation of interleukin‐6 production, kinase regulator activity, JAK–STAT signaling, and homotypic cell–cell adhesion (Figure 4I–N). To further characterize the biological relevance of the 53 intersecting targets, GO enrichment analysis was implemented with the help of Metascape. The top 10 entries for BP, CC, and MF were ranked by *p*‐value and visualized (Figure 4O). GO terms were primarily related to responses to lipopolysaccharide, peptide stimuli, and cellular secretion. Key cellular components included the presynaptic membrane, mitochondrial membrane, and extrinsic membrane components. Molecular functions involved collagen binding, protein kinase binding, and ATPase binding. In addition, KEGG pathway enrichment analysis revealed 35 significantly enriched signaling pathways. The top 10 pathways were visualized based on significance ranking (Figure 4P). Notably, Neuroactive ligand‐receptor interaction, Apoptosis, Rap1 signaling, and cAMP signaling pathways were among the most relevant to Vin‐mediated therapeutic regulation in ICH. Considering that the core targets of high value include JAK2, JAK1, JAK3, and MMP9, which are closely related to the JAK–STAT pathway (Table 2). Additionally, based on GeneMANIA, KEGG, and GO analyses, Vinorine was found to be associated with tyrosine protein phosphorylation, kinase regulator activity, receptor signaling pathway via JAK–STAT, and protein kinase binding in improving secondary brain damage following ICH. Combining this with previous research findings, we ultimately selected the JAK–STAT pathway as the pathway mechanism for further study.

### 3.4 Molecular Docking and MD Simulations of Core Target Proteins in Vin‐Mediated ICH Treatment

**FIGURE 5:**
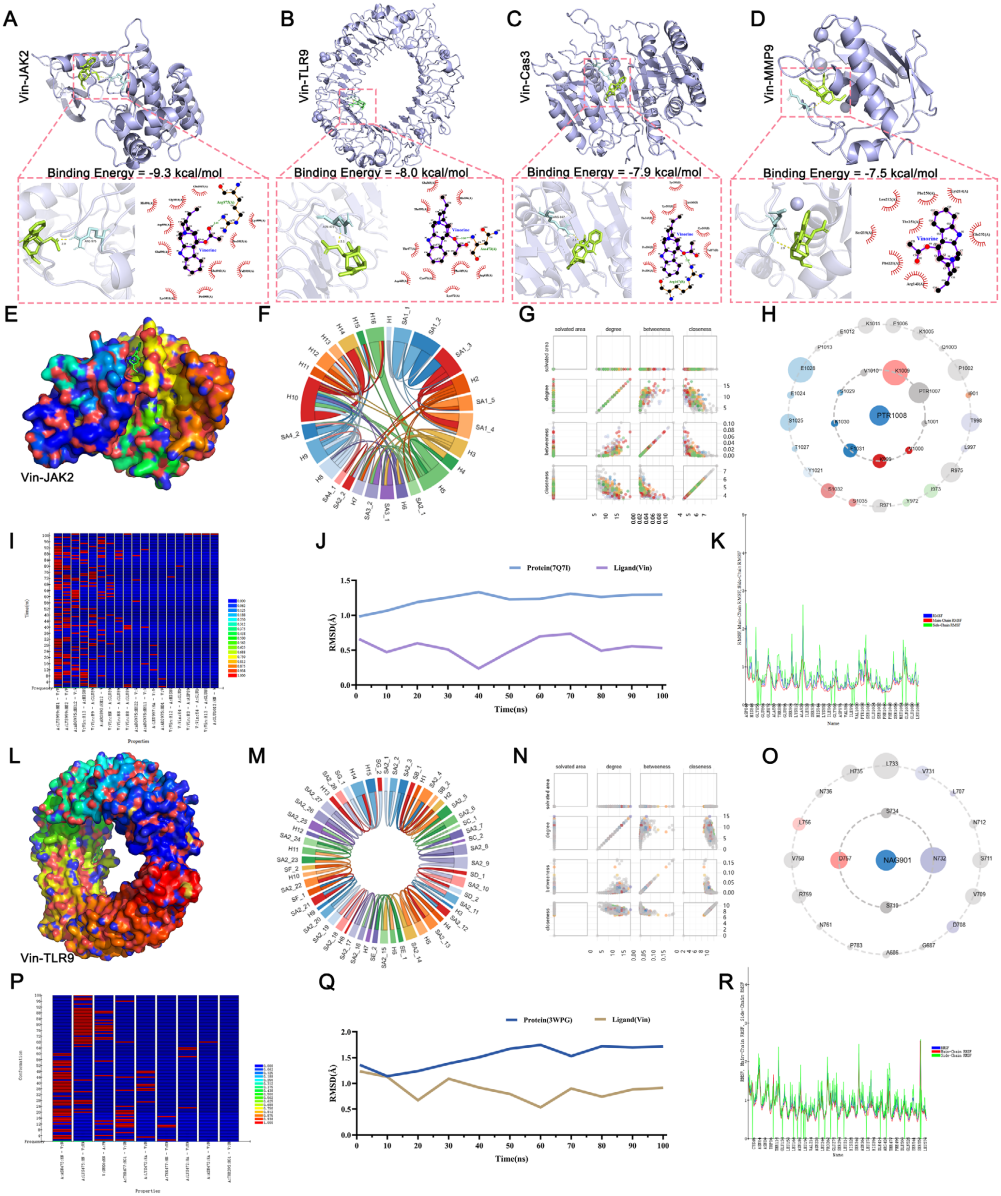
Molecular docking and molecular dynamics simulations of key target proteins with Vinorine. (A) Schematic diagram of Vinorine binding to JAK2. (B) Schematic diagram of Vinorine binding to TLR9. (C) Schematic diagram of Vinorine binding to Caspase3. (D) Schematic diagram of Vinorine binding to MMP9. (E) Specific binding pocket sites of Vinorine with JAK2. (F, G) Schematic diagram of amino acid residues bound by JAK2 inhibitors. (H) Amino acid asteroid plot of JAK2 inhibitor binding. The closer to the inner circle, the more critical the amino acid. (I) Schematic diagram of hydrogen bond changes within 100 ns after Vinorine binds to JAK2. (J) Changes in root mean square deviation (RMSD) for Vinorine and JAK2 upon binding, with an average RMSD of less than 1.5 Å. (K) Changes in RMSD of the main chain of the Vinorine‐JAK2 ligand‐protein complex. (L) Specific binding pocket site of Vinorine with TLR9. (M, N) Schematic diagram of TLR9 inhibitor binding residues. (O) Amino acid asteroid plot of TLR9 inhibitor binding. The closer to the inner circle, the more critical the amino acid. (P) Schematic diagram of hydrogen bond changes within 100 ns after Vinorine binds to TLR9. (Q) Changes in the root mean square deviation of Vinorine and TLR9 upon binding, with an average deviation of less than 2.0 Å. (R) Changes in the root mean square deviation of the main chain of the Vinorine‐TLR9 ligand‐protein complex.

To investigate the interaction between Vin and key ICH‐related targets, molecular docking was performed for four core proteins: JAK2, TLR9, Caspase‐3 (CASP3), and MMP9. AutoDock Vina results showed that Vin exhibited low binding energy values with all four targets (each below −5.0 kcal/mol), suggesting favorable and spontaneous binding interactions (Table 4). These findings support a strong affinity of Vin for critical proteins involved in ICH pathophysiology. The lowest‐energy docking conformations were visualized using PyMOL (Figure 5A–D), revealing specific spatial interactions between Vin and each protein. Among the targets, JAK2 exhibited both the highest degree value in the network and the strongest binding affinity to Vin (Table 4 and Figure 5E,L). To further characterize the interaction between Vin and JAK2, a residue‐level schematic of the JAK2 structure was generated (Figure 5F,G), and predicted interaction sites were compared with known inhibitor binding regions (Figure 5H). Molecular docking indicated that ARG975 is a likely critical residue for Vin binding, corresponding to residue R975 in the inhibitor binding site. Similarly, for TLR9, docking predicted a key interaction with ASN473, while the inhibitor‐based interaction map identified ASN732.

**TABLE 4:** The binding energy of compound and core targets (kcal/mol).

Target	PDB ID	Target structure	Compound	Affinity (kcal/mol)
JAK2	7Q7I	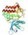	Vinorine	−9.3
TLR9	3WPG	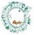	Vinorine	−8.0
MMP9	6ESM	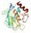	Vinorine	−7.9
Caspase 3	5JFT	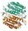	Vinorine	−7.5

To assess the stability and dynamic behavior of the ligand‐protein complexes, 100 ns MD simulations were implemented utilizing Discovery Studio 2019. For the Vin‐JAK2 complex, hydrogen bond dynamics revealed key interactions with residues LYS999, ARG975, ARG893, and LEU997, which varied over time (Figure 5I). JAK2 reached equilibrium around 50 ns, with an average RMSD of 1.27 Å, while Vin exhibited a lower average RMSD of 0.48 ± 0.18 Å (Figure 5J). Final RMSD values remained below 2.5 Å, confirming stable complex formation without major structural fluctuations (Figure 5J). RMSF analysis of the JAK2 backbone showed fluctuations within 0–1.5 Å, indicating minimal structural deviation under simulated physiological conditions (Figure 5K). For the Vin‐TLR9 complex, hydrogen bonding interactions primarily involved ASN473, LYS475, THR477, and LYS472, showing time‐dependent dynamics over the simulation period (Figure 5P). TLR9 reached dynamic equilibrium around 80 ns, with an average RMSD of 1.27 Å, while Vin's RMSD remained lower at 0.89 ± 0.34 Å (Figure 5Q). RMSF values for TLR9 ranged between 0 and 2.5 Å, indicating structural stability (Figure 5R). Collectively, these findings confirm that Vin forms stable complexes with both JAK2 and TLR9, with stronger and more stable binding observed for JAK2. Based on binding energy, docking conformation, and MD simulation data, JAK2 was validated as the primary molecular target of Vin in the context of ICH.

### 3.5 Vin Binds Tightly to the Pseudokinase Domain of JAK2 and Induces Conformational Changes

**FIGURE 6:**
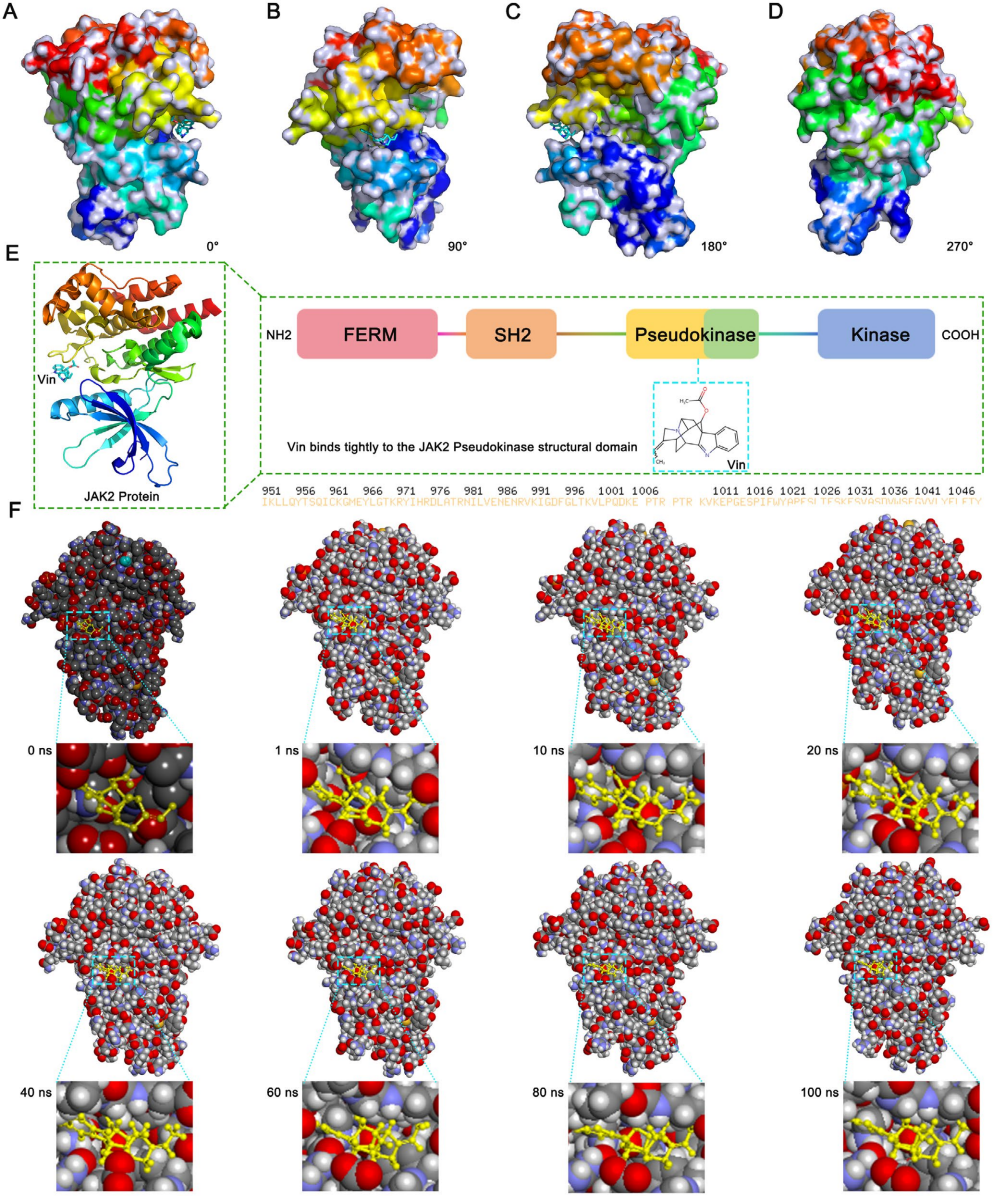
Investigation of the specific binding site between Vinorine and JAK2 and validation of their binding capacity. (A–D) Multi‐angle observation of the binding site between Vinorine and JAK2 (0°–270°). (E) Detailed analysis of the specific binding site between Vinorine and JAK2. Different colors correspond to different domains. (F) Representative conformational changes of the Vinorine‐JAK2 ligand‐protein complex within 100 ns.

To validate the interaction between Vin and JAK2, we analyzed the spatial localization of Vin within the JAK2 protein from multiple structural perspectives (Figure 6A–D). In a previous study, we detailed the domain architecture of JAK family proteins, comprising the FERM, SH2, pseudokinase, and kinase domains [22]. Notably, the pseudokinase domain shares structural similarities with the kinase domain but primarily functions as a regulatory module for kinase activation. Our docking analysis revealed that Vin binds specifically within the pseudokinase domain of JAK2 (Figure 6E). Given the regulatory role of this domain, Vin's interaction may modulate downstream phosphorylation events by influencing conformational dynamics within the kinase domain—ultimately affecting STAT protein activation. To assess conformational shifts during ligand binding, we performed MD simulations to monitor the spatial repositioning of Vin within JAK2 over a 100 ns time frame. As shown in Figure 6F, Vin underwent multiple conformational adjustments around its rotatable bonds while maintaining tight insertion into the pseudokinase pocket. Simultaneously, JAK2 displayed dynamic alterations in its surface side chains, consistent with conformational flexibility during ligand engagement. Despite these fluctuations, Vin remained stably embedded within the pseudokinase domain throughout the simulation, supporting a sustained binding interaction (Figure 6F).

### 3.6 Vin Affects ICH Prognostic Outcomes Through the CXCR2‐JAKs‐STATs Pathway

**FIGURE 7:**
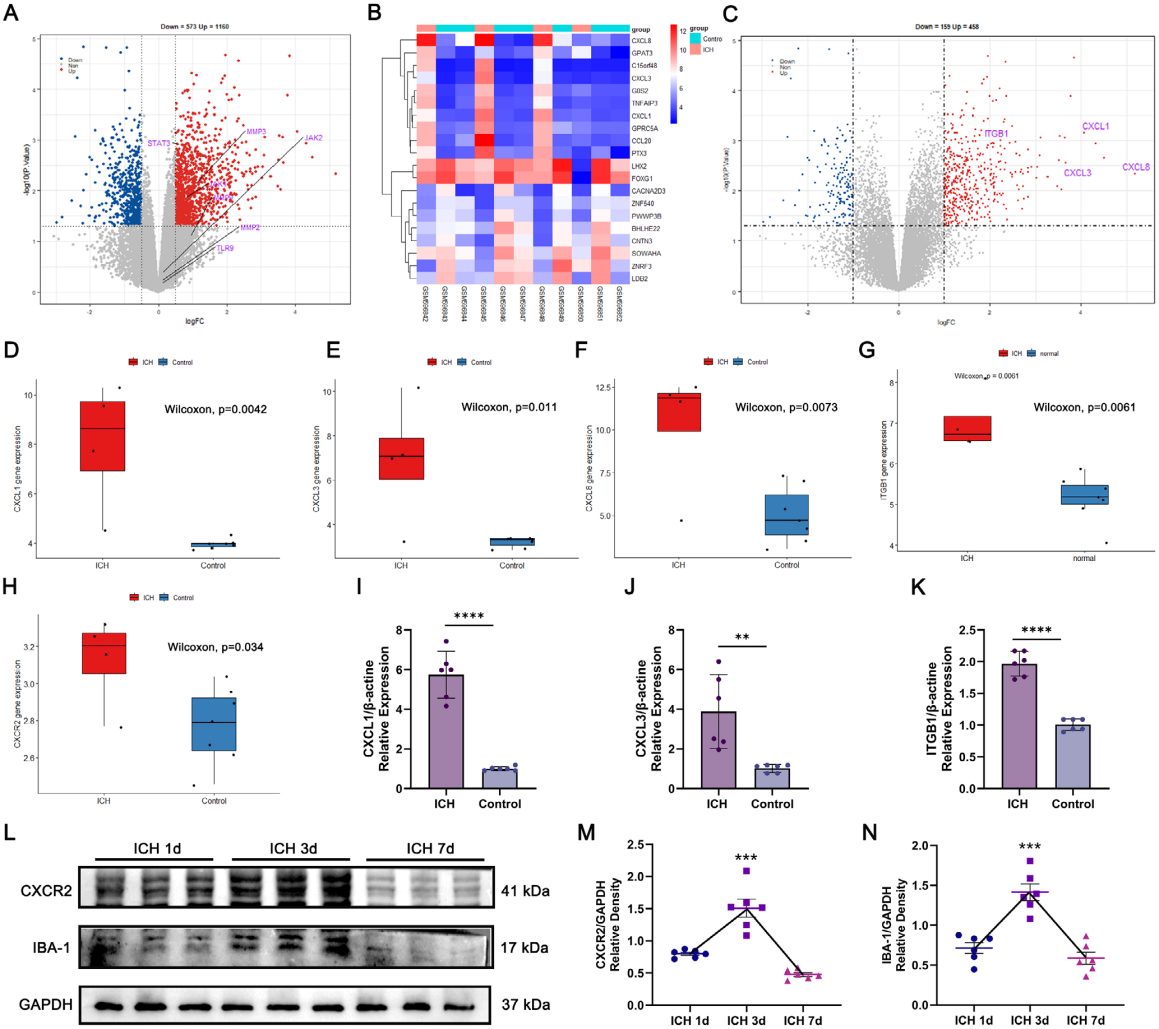
Clinical data analysis using DEGs reveals the association between the JAK–STAT pathway and ICH patients and analyses possible upstream and downstream proteins. (A) Volcano plot for |logFC| ≥ 0.5 and *p*‐value < 0.05, with the positions of target proteins such as JAK2, JAK1, STAT3, and MMPs annotated. (B) Heatmap of the top 20 differentially expressed genes in the intracerebral hemorrhage dataset. (C) Volcano plot for |logFC| ≥ 1.0 and *p*‐value < 0.05, with annotation of chemokine‐related proteins such as CXCL1, CXCL3, and CXCL8. (D–H) Violin plots showing the expression levels of CXCL1, CXCL3, CXCL8, ITGB1, and CXCR2 in the GSE24265 intracerebral hemorrhage dataset. (I–K) Q‐PCR detection of the expression of CXCL1, CXCL3, and ITGB1 in mouse brain tissue 3 days after ICH (*n* = 6). (L) Western blot experiment to detect the expression of CXCR2 and IBA‐1 proteins after ICH (*n* = 6). (M, N) Quantitative analysis of CXCR2 and IBA‐1 expression (*n* = 6). All data were expressed as mean ± standard deviation (SD). Statistical significance was determined by two‐way analysis of variance (ANOVA) and Tukey's multiple comparisons test, **p < 0.01, ***p < 0.001 and ****p < 0.0001 VS ICH group, *n* ≥ 6. ns, not significant.

To probe into the mechanism by which Vin improves prognostic outcomes in ICH, we analyzed differentially expressed genes (DEGs) from the GSE24265 dataset retrieved from the GEO database. The dataset comprised 15,271 genes, of which 1733 were identified as differentially expressed. Among these, 1160 genes were significantly upregulated, and 573 were downregulated, as visualized in the volcano plot (Figure 7A). Notably, JAK1, STAT3, and MMP3 were among the significantly upregulated genes. Previous studies have documented that activation of the JAK–STAT–MMPs axis is implicated in the pathophysiology surrounding hematomas after ICH, where excessive MMP expression disrupts BBB integrity and promotes neuronal apoptosis [37]. Given that JAK2, MMP9, and Caspase‐3 were identified as core Vin targets and combined with findings from GO, KEGG, and GeneMANIA analyses, we hypothesized that Vin may exert therapeutic effects by modulating this signaling pathway. To explore this further, we examined the top 20 DEGs via a heatmap. Interestingly, CXCL8, CXCL3, and CXCL1—all members of the CXC chemokine family—were highly upregulated (Figure 7B), a finding further confirmed by volcano and violin plots (Figure 7C–F). These results suggest that ICH induces elevated expression of chemokines around the hematoma in clinical patients. CXCLs are known to act via the CXCR2 receptor to regulate inflammation and immune cell recruitment, particularly of neutrophils and macrophages, often through STAT3‐mediated pathways [48]. Since leukocyte migration also depends on integrins, it is notable that ITGB1 expression was also significantly elevated in DEGs (Figure 7C,G). Violin plot analysis further indicated significantly increased CXCR2 expression following ICH (Figure 7H). These transcriptomic results were validated using qPCR and Western blot. qPCR confirmed upregulation of CXCL1, CXCL3, and ITGB1 in perihematomal brain tissue in ICH mice (Figure 7I–K). Western blot analysis revealed dynamic changes in CXCR2 protein expression over time (days 1, 3, and 7 post‐ICH) (Figure 7H), with peak expression observed on day 3 (Figure 7L,M). Notably, a similar temporal expression pattern was observed for the microglial marker IBA‐1 (Figure 7L,N). Collectively, these findings indicate that the CXCR2–JAKs–STATs–MMPs signaling may be activated by immune cells—particularly microglia—around the hematoma following ICH. Vin likely modulates this inflammatory pathway by targeting JAK2.

**FIGURE 8:**
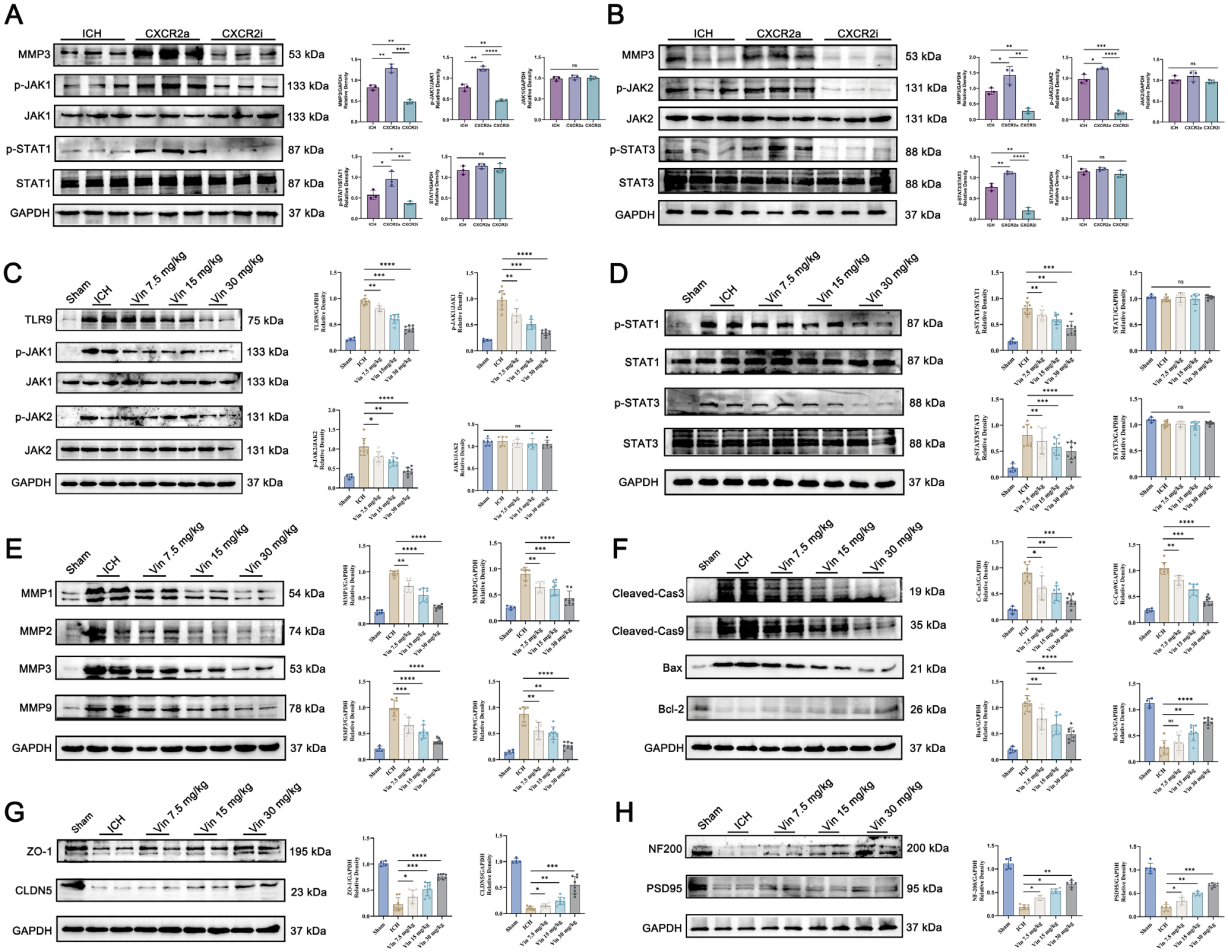
Expression of the CXCR2‐JAK–STAT signaling pathway around the haematoma after ICH, and the effects of Vinorine on the JAK–STAT signaling pathway around the haematoma, brain tissue apoptosis, and blood–brain barrier function. (A, B) Western blot experiments confirmed the activation of the CXCR2‐mediated JAK–STAT signaling pathway around the haematoma (*n* = 6). (C, D) Western blot experiments detected the effects of Vinorine on the expression of JAK–STAT signaling pathway‐related proteins in the perihaematoma region. (E) Western blot experiments detected the effects of Vinorine on the expression of MMPs in the perihaematoma region. (F) Western blot experiments detected the effects of Vinorine on the expression of apoptosis‐related proteins such as BCL‐2, Cleaved‐Caspase 3, and Cleaved‐Caspase 9 in the perihaematoma region. (G) Western blot experiments were conducted to assess the effects of Vinorine on the expression of blood–brain barrier proteins ZO‐1 and CLDN5. (H) Western blot experiments were conducted to assess the effects of Vinorine on the expression of neuronal axonal proteins NF200 and PSD95. All data were expressed as mean ± standard deviation (SD). Statistical significance was determined by two‐way analysis of variance (ANOVA) and Tukey's multiple comparisons test, *p < 0.05, **p < 0.01, ***p < 0.001 and # or ****p < 0.0001 VS ICH group, *n* ≥ 4. ns, not significant.

To further validate the involvement of the CXCR2‐JAKs‐STATs signaling pathway in ICH, we examined the expression levels of p‐JAK1, p‐JAK2, p‐STAT1, and p‐STAT3 via Western blot analysis following the administration of a CXCR2‐specific agonist or inhibitor. As depicted in Figure 8A,B, treatment with a CXCR2 agonist notably increased the expression of all four phosphorylated proteins in the perihematomal region. Conversely, administration of a CXCR2 inhibitor resulted in a marked reduction in phosphorylation levels. These results confirm that the CXCR2‐JAK–STAT axis is activated after ICH onset and may contribute to the disease's progression. We next evaluated the effect of Vin treatment on this signaling cascade. Compared to the sham group, ICH induced significant activation of the JAK–STAT‐MMP pathway, as evidenced by elevated levels of all four phosphorylated proteins, along with increased expression of downstream MMPs. Notably, Vin treatment attenuated this activation in a dose‐dependent fashion (Figure 8C–E), suggesting that its neuroprotective effects are closely tied to the inhibition of this inflammatory and tissue‐degrading pathway. In addition, Western blot analysis demonstrated that Vin notably reduced the expression of pro‐apoptotic markers, including cleaved Caspase‐3/9 and Bax, in perihematomal brain tissue (Figure 8F), indicating a protective effect against ICH‐induced neuronal apoptosis. Furthermore, Vin treatment preserved BBB integrity, as shown by reduced degradation of ZO‐1 and CLDN5 tight junction proteins following ICH (Figure 8G). This BBB protection was accompanied by enhanced expression of neuronal axonal proteins, such as NF200 and PSD95 (Figure 8H).

### 3.7 Effect of Vin on the JAK–STAT Pathway and Polarization of Hemin‐Pretreated BV2 Cells

**FIGURE 9:**
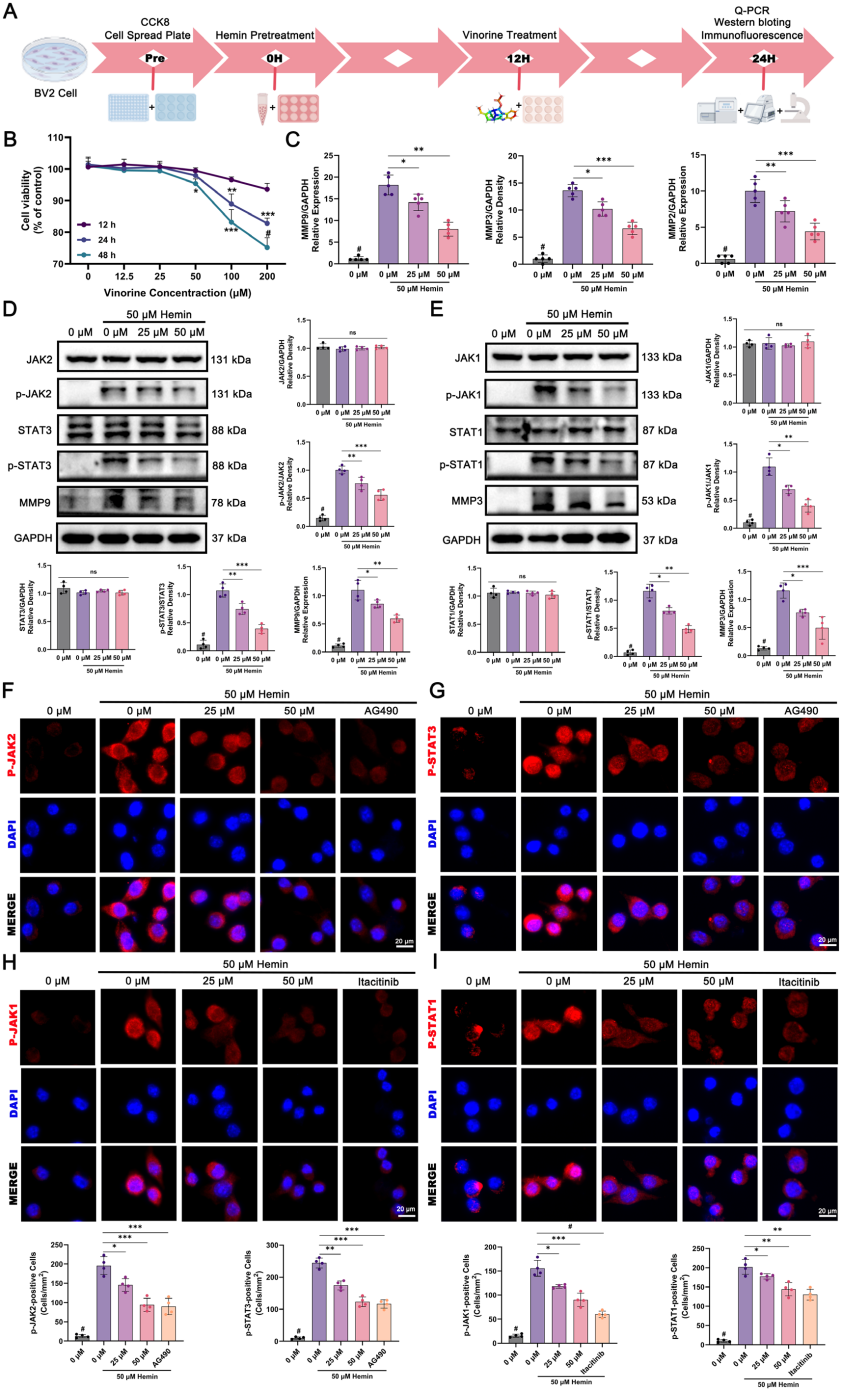
In vitro experiments validate the effect of Vinorine on the JAK–STAT signaling pathway in Hemin‐pretreated BV2 cells. (A) Timeline diagram of BV2 cells used in the in vitro study. (B) Screening of the optimal dosage concentration and treatment time of Vinorine through the CCK8 assay. (C) Detection of MMP9, MMP3, and MMP2 levels in BV2 cells from each group after hemin pretreatment via q‐PCR. (D) Western blot analysis of Vinorine's effects on the expression levels of JAK2‐STAT3 pathway‐related proteins in BV2 cells after Hemin pretreatment. (E) Western blot analysis of Vinorine's effects on the expression levels of JAK1‐STAT1 pathway‐related proteins in BV2 cells after Hemin pretreatment. (F, G) Immunofluorescence detection of p‐JAK2 and p‐STAT3 protein expression in BV2 cells after hemin pretreatment. Scale bar = 20 μm. (H, I) Immunofluorescence detection of p‐JAK1 and p‐STAT1 protein expression in BV2 cells after hemin pretreatment. Scale bar = 20 μm. All data were expressed as mean ± sandard deviation (SD). Statistical significance was determined by two‐way analysis of variance (ANOVA) and Tukey's multiple comparisons test, *p < 0.05, **p < 0.01, ***p < 0.001 and # VS 0 μM (50 μM Hemin pre‐treated) group, *n* ≥ 4. ns, not significant.

Considering the central role of the JAK–STAT pathway in immune signaling and the immune function of microglia, we examined Vin's impact on this pathway in BV2 cells (Figure 9A). BV2 cells were first treated with various concentrations of Vin (0, 12.5, 25, 50, 100, and 200 μM) for 12, 24, and 48 h, followed by cell viability assessment. As determined by the CCK‐8 assay (Figure 9B), 25 μM and 50 μM concentrations were selected for subsequent experimentations. To model ICH in vitro, BV2 cells were pretreated with 50 μM Hemin for 12 h. qPCR demonstrated that Hemin treatment distinctly upregulated the expression of MMP9, MMP3, and MMP2—downstream genes of the JAK–STAT signaling axis. Vin treatment dose‐dependently suppressed this upregulation (Figure 9C). Western blot analysis further confirmed the molecular findings (Figure 9D,E). To visualize these molecular changes, cellular immunofluorescence staining was performed. In addition to Vin treatment, two known JAK pathway inhibitors—AG490 (JAK2 inhibitor) and Itacitinib (JAK1 inhibitor)—were used as controls. Compared with untreated controls, Hemin exposure led to increased expression of p‐JAKs and p‐STATs, which was significantly attenuated by both Vin and the inhibitors, particularly at 50 μM Vin (Figure 9F–I). Importantly, nuclear translocation of p‐STATs, a hallmark of the JAK–STAT pathway activation, was observed after Hemin treatment. Vin markedly reduced this nuclear localization, indicating effective suppression of the JAK–STAT signaling activation (Figure 9G,I). In addition, AG490 treatment significantly decreased p‐JAK2 and p‐STAT3 expression, with a reduction comparable to that achieved by 50 μM Vin, suggesting that Vin may exert similar inhibitory effects as AG490. A similar pattern was observed with Itacitinib, which targets JAK1, reinforcing the non‐selective inhibitory potential of Vin across JAK family members (Figure 9F–I). These findings support the hypothesis that Vin binds to a conserved structural region in the kinase or pseudokinase domain shared by JAK1, JAK2, and possibly JAK3, resulting in broad suppression of JAK–STAT phosphorylation. This observation aligns with earlier network pharmacology predictions and experimental data indicating that multiple JAK isoforms are among Vin's predicted therapeutic targets in ICH.

**FIGURE 10:**
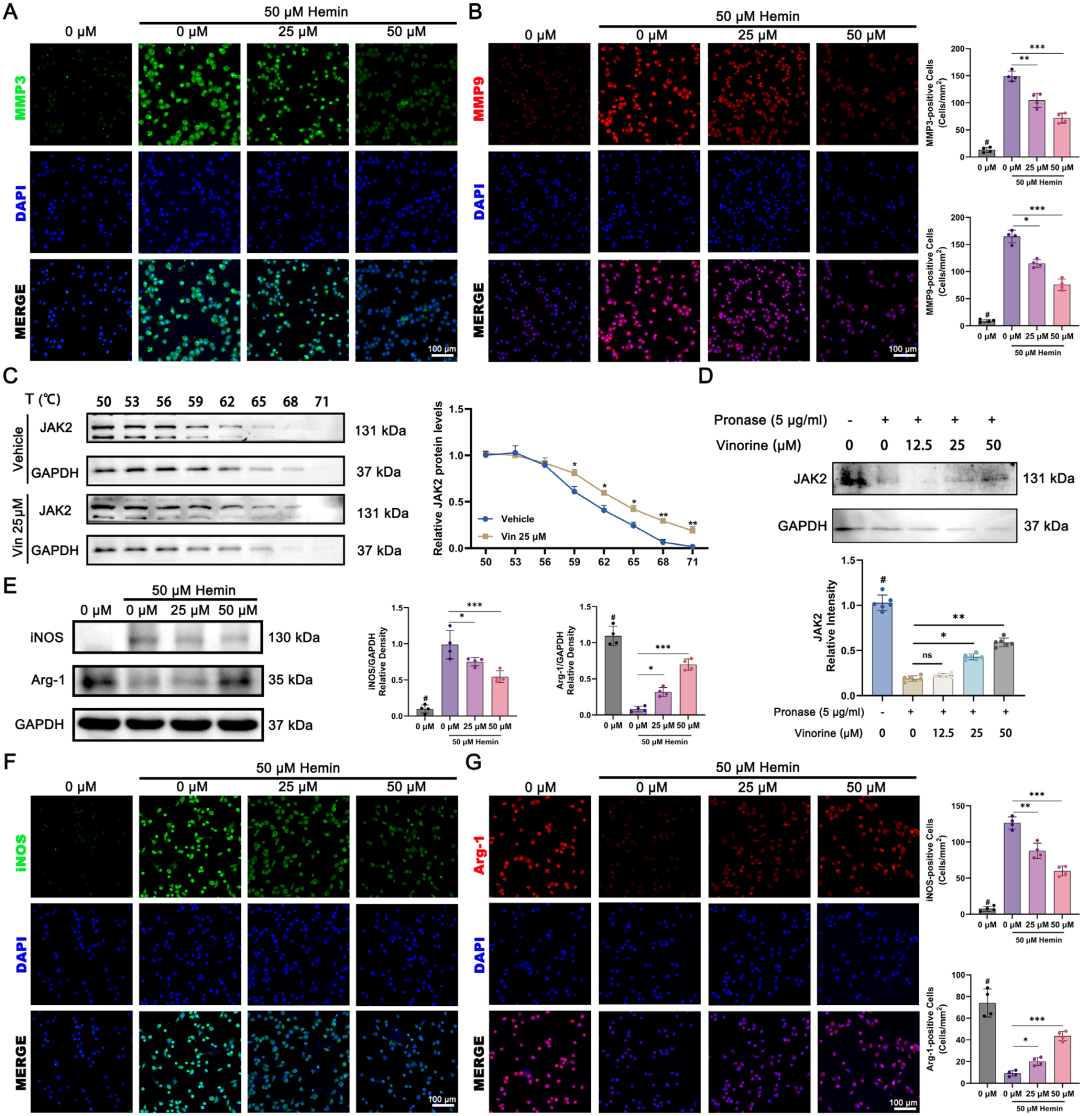
In vitro experiments to assess the effects of Vinorine on MMP expression and cell polarization in Hemin‐pretreated BV2 cells. (A) Immunofluorescence detection of MMP3 expression in Hemin‐pretreated BV2 cells treated with Vinorine. Scale bar = 100 μm. (B) Immunofluorescence detection of MMP9 expression in Hemin‐pretreated BV2 cells treated with Vinorine. Scale bar = 100 μm. (C) CETSA experiment to assess the heat resistance of the Vinorine‐JAK2 binding complex. (D) DARTS experiment to assess the enzymatic stability of the Vinorine‐JAK2 binding complex. (E) Western blot analysis of iNOS and Arg‐1 expression levels in BV2 cells from each group after hemin pretreatment. (F) Immunofluorescence analysis of Vinorine's effect on iNOS (M1 marker, green) expression in BV2 cells after hemin pretreatment. Scale bar = 100 μm. (G) Immunofluorescence detection of Arg‐1 (M2‐type marker, red) expression in BV2 cells after hemin pretreatment. Scale bar = 100 μm. All data were expressed as mean ± standard deviation (SD). Statistical significance was determined by two‐way analysis of variance (ANOVA) and Tukey's multiple comparisons test, *p < 0.05, **p < 0.01, ***p < 0.001 and # VS 0 μM (50 μM Hemin pre‐treated) group, *n* ≥ 4. ns, not significant.

To directly evaluate the effect of Vin on MMP expression in BV2 microglial cells following Hemin exposure, we performed immunofluorescence staining for MMP3 and MMP9. The results showed that Hemin treatment evidently upregulated MMP3 and MMP9 expression in BV2 cells, whereas Vin administration markedly reduced the expression of both proteins (Figure 10A,B). To assess the stability of Vin binding to JAK2, we performed CETSA and DARTS. As illustrated in Figure 10C,D, the presence of Vin significantly enhanced the thermal stability and protease resistance of the JAK2 protein, indicating the formation of a stable Vin‐JAK2 complex. Collectively, Vin directly interacts with JAK2 in BV2 cells, validating the computational predictions and further supporting the biological relevance of JAK2 as a primary target of Vin in ICH. To assess the impact of Vin on microglial polarization, we conducted a Western blot analysis for polarization markers. Hemin pretreatment resulted in a notable elevation in iNOS and a decline in Arg‐1. Vin treatment effectively reversed these changes, showing a dose‐dependent decrease in iNOS and increase in Arg‐1 expression (Figure 10E). Consistent with the Western blot findings, immunofluorescence staining also demonstrated that Vin significantly suppressed iNOS expression and enhanced Arg‐1 expression in BV2 cells compared with Hemin treatment alone. These effects were most pronounced at a concentration of 50 μM Vin (Figure 10F,G). Collectively, Vin effectively inhibits the JAK–STAT‐MMPs signaling cascade and promotes anti‐inflammatory (M2‐like) polarization of BV2 microglial cells following Hemin‐induced injury.

### 3.8 Effect of Vin on HT22 Cells Pretreated With Hemin

**FIGURE 11:**
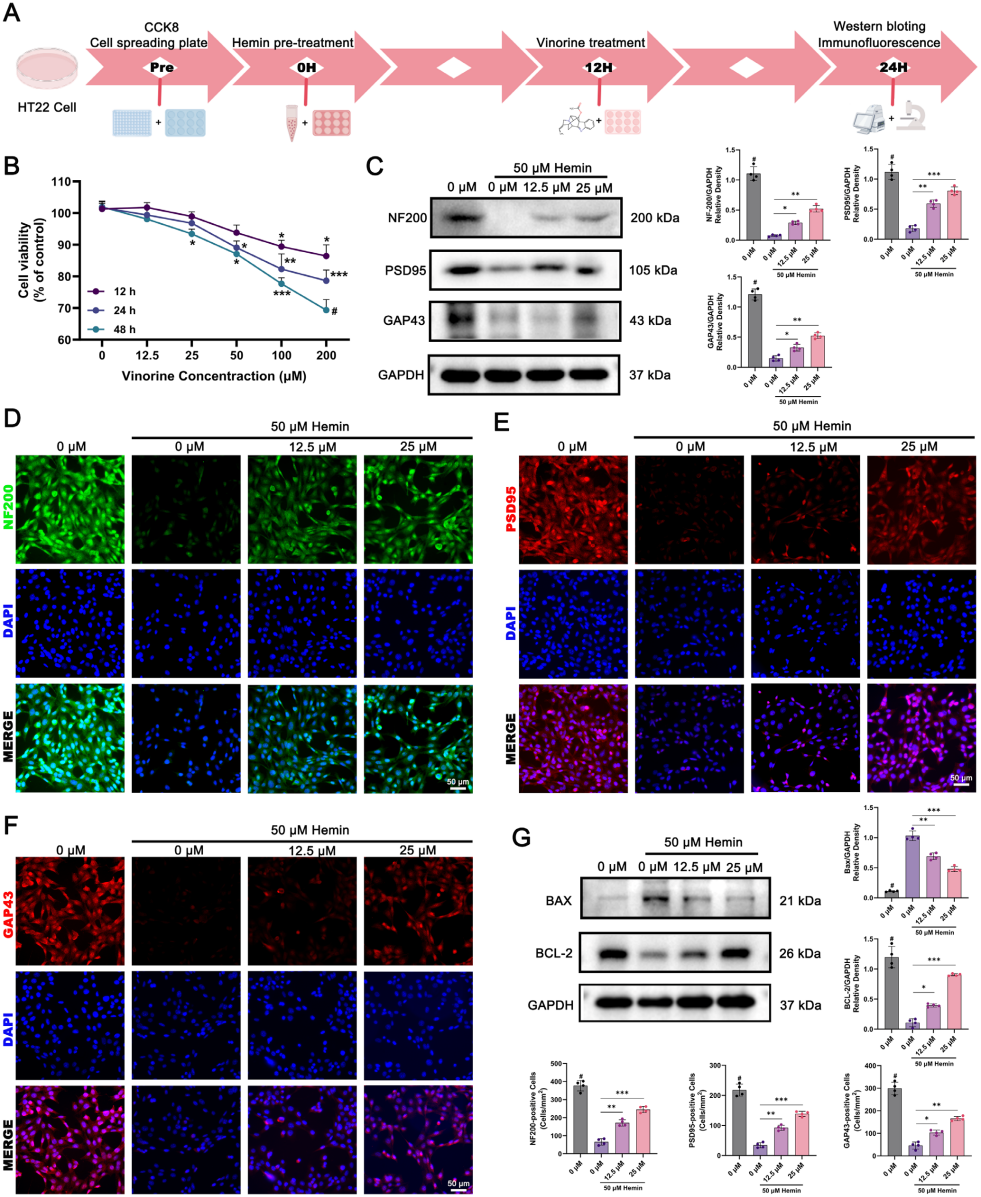
In vitro experiments to validate the effect of Vinorine on Hemin‐pretreated HT22 cells. (A) Timeline diagram of HT22 cells used in the in vitro study. (B) CCK8 assay used to screen for the optimal Vinorine dosage and treatment time. (C) Western blot analysis of NF200, PSD95, and GAP43 expression levels in HT22 cells from each group after hemin pretreatment (*n* = 4). (D) Immunofluorescence detection of NF200 protein expression in Hemin‐pretreated HT22 cells treated with Vinorine. Scale bar = 50 μm. (E) Immunofluorescence detection of PSD95 protein expression in Hemin‐pretreated HT22 cells treated with Vinorine. Scale bar = 50 μm. (F) Immunofluorescence detection of GAP43 protein expression in HT22 cells after Hemin‐pretreatment. Scale bar = 50 μm. (G) Western blot analysis of Bax and Bcl‐2 expression levels in HT22 cells after Hemin pretreatment (*n* = 4). All data were expressed as mean ± standard deviation (SD). Statistical significance was determined by two‐way analysis of variance (ANOVA) and Tukey's multiple comparisons test, *p < 0.05, **p < 0.01, ***p < 0.001 and # VS 0 μM (50 μM Hemin pre‐treated) group, *n* ≥ 4. ns, not significant.

Given that Vin treatment in this study was administered after ICH onset, neurons would initially be exposed to erythrocyte lysis products—such as Hemin—prior to encountering Vin. To mimic this pathological sequence in vitro, we established a cellular ICH model using 50 μM Hemin‐pretreated HT22 mouse hippocampal neuronal cells, followed by Vin administration (Figure 11A). Initially, HT22 cells were treated with varying concentrations of Vin, followed by cell viability assessment. It was evident that (Figure 11B), 12.5 μM and 25 μM were selected as standard concentrations for subsequent experiments. To evaluate the neuroprotective effect of Vin following Hemin‐induced injury, Western blot analysis was performed. Hemin treatment alone significantly reduced the expression of axonal structural proteins—NF200, PSD95, and GAP43—in HT22 cells. However, Vin administration restored the expression of these proteins in a dose‐dependent fashion, with 50 μM Vin showing the most pronounced effect (Figure 11C). These results were further corroborated by immunofluorescence staining (Figure 11D–F). Furthermore, Western blot analysis showed that Vin notably reduced neuronal apoptosis in Hemin‐pretreated HT22 cells, indicating a protective effect against erythrocyte lysis product‐induced cytotoxicity (Figure 11G). In conclusion, these results suggest that Vin mitigates Hemin‐induced neuronal injury by promoting axonal protein expression and suppressing apoptosis.

## 4 Discussion

ICH, defined as spontaneous bleeding within brain tissue without trauma [49], is often linked to severe outcomes. Currently, no effective pharmacological treatments have been approved for ICH, underscoring the urgent need for novel therapeutic strategies. Post‐ICH injury involves a cascade of complex pathophysiological events, including immune‐inflammatory responses, neuronal apoptosis, and BBB disruption [50]. However, the precise molecular mechanisms underlying secondary brain injury following ICH remain incompletely understood. Emerging evidence suggests that aberrant activation of the JAK–STAT axis is a key contributor to the pathogenesis and poor prognosis of ICH [27].

In this study, we identified Vin, a monoterpene indole alkaloid derived from plants of the *Apocynaceae* family, as a promising therapeutic candidate for ICH. Our in vivo results demonstrated that Vin evidently improved functional recovery in ICH mice, including enhanced limb coordination, reduced neurological deficits, and decreased hematoma volume. Additionally, Vin alleviated cerebral edema, preserved BBB integrity, mitigated neuronal damage, and promoted long‐term neurofunctional recovery. To elucidate the underlying mechanisms, we combined network pharmacology, molecular docking, MD simulations, and experimental validation. JAK2 emerged as a key molecular target of Vin. Further pathway analysis—including GO, KEGG, and differential gene expression profiling—revealed that Vin modulates the CXCR2‐JAKs‐STATs axis, predominantly by inhibiting JAK phosphorylation.

Microglia, the resident immune cells of the central nervous system, are the first non‐neuronal cells to respond following ICH [51]. Representing approximately 5%–10% of all brain cells, microglia are critical in mediating the immune‐inflammatory response and facilitating hematoma clearance after ICH [52]. Notably, microglial activation can persist for up to 3 months post‐ICH. During the acute phase of injury, “pro‐inflammatory” (M1‐type) microglia accumulate around the hematoma and exacerbate inflammation by activating the JAK–STAT pathway, which promotes the secretion of MMPs. This contributes to BBB disruption and neuronal apoptosis [53]. In contrast, during the chronic recovery phase, “anti‐inflammatory” (M2‐type) microglia aid in hematoma resolution and tissue repair by phagocytosing cellular debris and engaging in receptor‐mediated clearance via CD163, CD36, and CD47 [54]. Therapeutically promoting the phenotypic switch from M1 to M2 microglia represents a promising strategy for both anti‐inflammatory modulation and hematoma absorption in ICH. iNOS and Arg‐1 serve as established markers for M1‐ and M2‐type microglia, respectively. In our study, Vin treatment notably diminished iNOS expression while upregulating Arg‐1 in microglia, suggesting that Vin facilitates microglial polarization toward the M2 phenotype. Moreover, inhibition of the JAK–STAT signaling in microglia following ICH has been linked to improved BBB integrity and reduced neuronal injury. Our findings further demonstrated that Vin markedly suppressed JAK phosphorylation in microglia, thereby attenuating downstream MMP expression. Collectively, these results indicate that Vin not only inhibits microglial‐mediated JAK–STAT pathway activation but also promotes a shift toward an anti‐inflammatory microglial phenotype, contributing to neuroprotection and hematoma resolution after ICH.

In the nervous system, the JAK–STAT pathway is closely linked to microglial function. This evolutionarily conserved pathway transduces extracellular cytokine signals—such as chemokines—into nuclear transcriptional responses via target gene regulation. Upon cytokine binding, transmembrane receptors undergo subunit dimerization, facilitating the recruitment and activation of JAKs through transphosphorylation and/or autophosphorylation [22]. Activated JAKs subsequently phosphorylate tyrosine residues on the intracellular domains of cytokine receptors. These phosphotyrosine motifs serve as docking sites for STATs through their Src homology 2 domains [55]. JAK‐mediated phosphorylation of STAT proteins at their C‐terminal domains leads to their dissociation from the receptor complex, followed by homo‐ or heterodimerization and nuclear translocation [56]. Within the nucleus, STAT dimers bind to specific DNA promoter elements, driving the transcription of downstream target genes involved in inflammatory and apoptotic processes, including MMPs, Bax, and Caspases [57].

In the present study, we employed network pharmacology, computational modeling, and both cellular and animal experimental approaches to test the therapeutic potential of Vin in ICH. The results validated that Vin significantly improved neurological outcomes following ICH. In vivo, this protective effect was attributed to Vin's ability to inhibit the CXCR2/JAK/STAT signaling, particularly through suppression of JAK2 phosphorylation in perihematomal regions—a process tightly linked to microglial activation. In vitro, Vin promoted the polarization of microglia from the M1 phenotype to the M2 phenotype and simultaneously preserved the expression of neuronal axonal proteins under injurious conditions. Notably, this study is the first to probe the significance of Vin in ICH‐induced neuroinflammation and neuronal injury, offering novel mechanistic insights into its therapeutic potential for hemorrhagic stroke. However, certain limitations should be acknowledged. Although our findings clearly demonstrate that Vin suppresses JAK2 and JAK1 phosphorylation and consequently reduces STAT activation, we did not in‐depth investigate its regulatory effects on other key members of the JAK–STAT pathway, such as STAT2 and STAT5 (Figure S3). Additionally, it is known that gender differences can influence stroke outcomes and immune responses. Hormonal differences between male and female mice may lead to varying responses to drug therapy following ICH. This study utilized only female mice, and it remains unclear whether Vin exhibits the same therapeutic effects in male mice. Second, while the current study highlights the binding of Vin to the JAK2 target protein, it does not explore the binding mode between the two or the amino acid binding sites through more in‐depth methods. These proteins also play critical roles in cytokine signaling and immune modulation. Therefore, further studies are warranted to comprehensively assess how Vin modulates the phosphorylation and activation of these additional components, in order to fully elucidate its mechanism of action in ICH.

## 5 Conclusion

In conclusion, this study systematically investigated the potential therapeutic targets and molecular mechanisms of Vin in ICH treatment through an integrated approach combining network pharmacology, bioinformatics, and computer‐aided prediction, along with in vivo animal experiments and in vitro cellular validation. The results demonstrated that Vin exerted neuroprotective effects in ICH primarily by targeting key proteins, including JAK2, TLR9, Caspase‐3, and MMP9, with JAK2 identified as a particularly crucial mediator. Mechanistically, our findings suggested that Vin alleviated ICH‐induced injury by modulating the CXCR2‐JAK–STAT axis, thereby attenuating neuroinflammation and promoting neural protection. Specifically, Vin suppressed the JAK–STAT pathway activation in microglial cells, resulting in decreased expression of MMPs, polarization of microglia toward an anti‐inflammatory (M2‐like) phenotype, and reduced neuroinflammatory damage. Moreover, in HT22 hippocampal neurons, Vin preserved axonal protein expression and inhibited Hemin‐induced apoptosis. These findings enhance our understanding of the molecular basis of Vin's therapeutic actions in ICH and underscore its potential as a candidate compound for pharmacological intervention in hemorrhagic stroke. However, given the complex and multifactorial nature of ICH pathophysiology, the precise molecular targets and signaling networks involved in Vin‐mediated protection require further elucidation. Future research should aim to dissect these mechanisms in greater detail and conduct preclinical translational studies to validate its efficacy and safety in clinically relevant models.

## Author Contributions

**Jia‐Wei Wu:** investigation, validation, visualization, writing – review and editing, software, writing – original draft. **Yi‐Ting Zhou:** validation, conceptualization, methodology, writing – review and editing. **Bing‐Xin Wang:** investigation, conceptualization, writing – review and editing. **Peng Wang:** writing – review and editing. **Xu‐Qi Zhang** and **Shi‐Qing Du:** validation, conceptualization. **Xiao‐Jie Lu:** conceptualization, project administration. **Zeng‐Li Miao:** funding acquisition, data curation, supervision. **Xu‐Dong Zhao:** conceptualization, funding acquisition, data curation, methodology, supervision.

## Ethics Statement

Institutional Review Board Statement: All experiments strictly followed the National Institutes of Health Guidelines for the Care and Use of Laboratory Animals and were approved by the laboratory animal welfare and ethics committee of Wuxi Medical College, Jiangnan University (approval number: JN.No20240515c0160472 [054]).

## Conflicts of Interest

The authors declare no conflicts of interest.

## Supporting information


**Figure S1:** The effects of Vinorine on mouse liver, kidney, and other organs. (A) Representative images of HE staining of liver, kidney, heart, spleen, and lung structures in different groups (*n* = 4). Scale bar = 50 μm. (B) Bar charts of serum levels of AST, ALT, UREA, CREA, LDH, and CK in different groups (*n* = 3). All data were expressed as mean ± standard deviation (SD). Statistical significance was determined by two‐way analysis of variance (ANOVA) and Tukey's multiple comparisons test, * *p* < 0.05, ** *p* < 0.01, *** *p* < 0.001 and # or **** *p* < 0.0001 VS ICH group, *n* ≥ 3. ns, not significant.
**Figure S2:** Evans blue and molecular docking related information. (A) Schematic diagram of Evans Blue dye staining of brain tissue. (B) Evans Blue‐related statistical chart. (C) Schematic diagram of the docking mode of Vinorine with related proteins. (D) SwissADME website prediction of Vinorine‐related information. All data were expressed as mean ± standard deviation (SD). Statistical significance was determined by two‐way analysis of variance (ANOVA) and Tukey's multiple comparisons test, * *p* < 0.05, ** *p* < 0.01, *** *p* < 0.001 and # or **** *p* < 0.0001 VS ICH group, *n* ≥ 4. ns, not significant.
**Figure S3:** Effects of Vinorine on other proteins in the JAK–STAT pathway and downstream proteins. (A) The effect of Vinorine on IL‐6 and SOCS3 proteins. (B) Effects of Vinorine on JAK3, TYK2, and STAT6 protein phosphorylation levels. (C) Validation of the efficacy of silent JAK2 protein plasmids. (D) Verification of the effects of Vinorine and JAK2 siRNA on JAK2 protein phosphorylation. (E, F) Verification of CXCR2‐JAK–STAT axis activation in BV2 cells. All data were expressed as mean ± standard deviation (SD). Statistical significance was determined by two‐way analysis of variance (ANOVA) and Tukey's multiple comparisons test, * *p* < 0.05, ** *p* < 0.01, *** *p* < 0.001 and # or **** *p* < 0.0001 VS 0 μM (50 μM Hemin pre‐treated) group, *n* ≥ 4. ns, not significant.

## Data Availability

The data support the findings of this study and are available from the corresponding author upon reasonable request. The relevant raw data can be found in: WU, JIAWEI; ZHAO, XUDONG (2025), “Vinorine targets the CXCR2‐JAK‐STAT axis to regulate microglial polarisation and alleviate secondary damage in Intracerebral hemorrhage”, Mendeley Data, V1, doi: 10.17632/r84sbmwsjk.1.
