# Supplementary material for: Network Pharmacology‐Based and Experimental Validation Elucidate the Target Mechanism of Vinorine in Ameliorating Secondary Brain Injury After Intracerebral Hemorrhage
Source: CNS Neurosci Ther. 2025 Sep 25;31(9):e70609. doi: 10.1111/cns.70609 (PMC12461119; doi:10.1111/cns.70609)
Supplement: Supplementary file 1 — Figure S1: The effects of Vinorine on mouse liver, kidney, and other organs. (A) Representative images of HE staining of liver, kidney, heart, spleen, and lung structures in different groups (n = 4). Scale bar = 50 μm. (B) Bar charts of serum levels of AST, ALT, UREA, CREA, LDH, and CK in different groups (n = 3). All data were expressed as mean ± standard deviation (SD). Statistical significance was determined by two‐way analysis of variance (ANOVA) and Tukey's multiple comparisons test, * p < 0.05, ** p < 0.01, *** p < 0.001 and # or **** p < 0.0001 VS ICH group, n ≥ 3. ns, not significant. Figure S2: Evans blue and molecular docking related information. (A) Schematic diagram of Evans Blue dye staining of brain tissue. (B) Evans Blue‐related statistical chart. (C) Schematic diagram of the docking mode of Vinorine with related proteins. (D) SwissADME website prediction of Vinorine‐related information. All data were expressed as mean ± standard deviation (SD). Statistical significance was determined by two‐way analysis of variance (ANOVA) and Tukey's multiple comparisons test, * p < 0.05, ** p < 0.01, *** p < 0.001 and # or **** p < 0.0001 VS ICH group, n ≥ 4. ns, not significant. Figure S3: Effects of Vinorine on other proteins in the JAK–STAT pathway and downstream proteins. (A) The effect of Vinorine on IL‐6 and SOCS3 proteins. (B) Effects of Vinorine on JAK3, TYK2, and STAT6 protein phosphorylation levels. (C) Validation of the efficacy of silent JAK2 protein plasmids. (D) Verification of the effects of Vinorine and JAK2 siRNA on JAK2 protein phosphorylation. (E, F) Verification of CXCR2‐JAK–STAT axis activation in BV2 cells. All data were expressed as mean ± standard deviation (SD). Statistical significance was determined by two‐way analysis of variance (ANOVA) and Tukey's multiple comparisons test, * p < 0.05, ** p < 0.01, *** p < 0.001 and # or **** p < 0.0001 VS 0 μM (50 μM Hemin pre‐treated) group, n ≥ 4. ns, not significant. [file CNS-31-e70609-s001.docx]

**Supplementary Material**

**
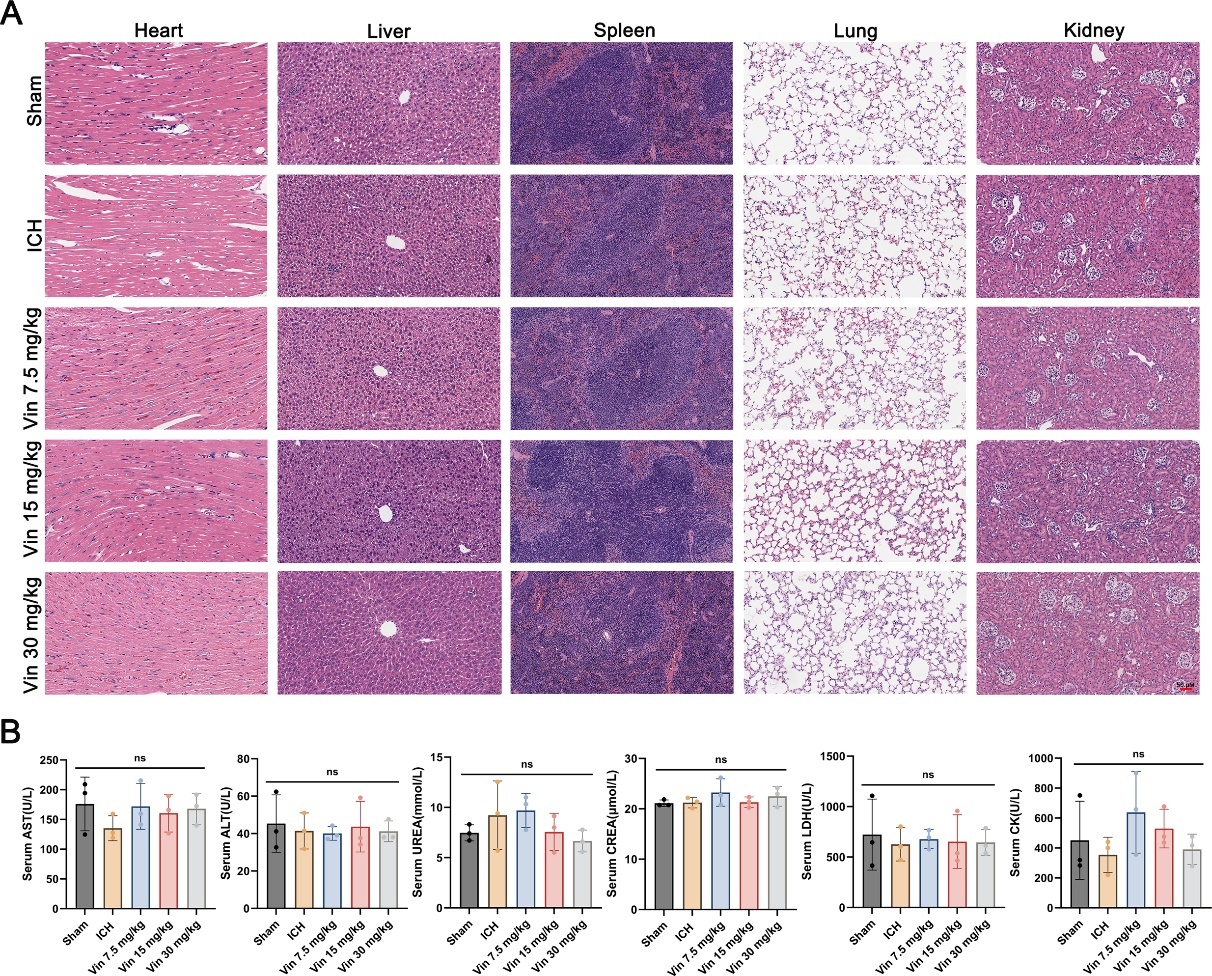
**

Figure S1. The effects of Vinorine on mouse liver, kidney, and other organs. (A) Representative images of HE staining of liver, kidney, heart, spleen, and lung structures in different groups (n=4). Scale bar = 50 μm. (B) Bar charts of serum levels of AST, ALT, UREA, CREA, LDH, and CK in different groups (n=3). All data were expressed as mean ± standard deviation (SD). Statistical significance was determined by two-way analysis of variance (ANOVA) and Tukey's multiple comparisons test, * p < 0.05, ** p < 0.01, *** p < 0.001 and ^#^ or **** p < 0.0001 VS ICH group, n ≥ 3. ns: not significant.

**
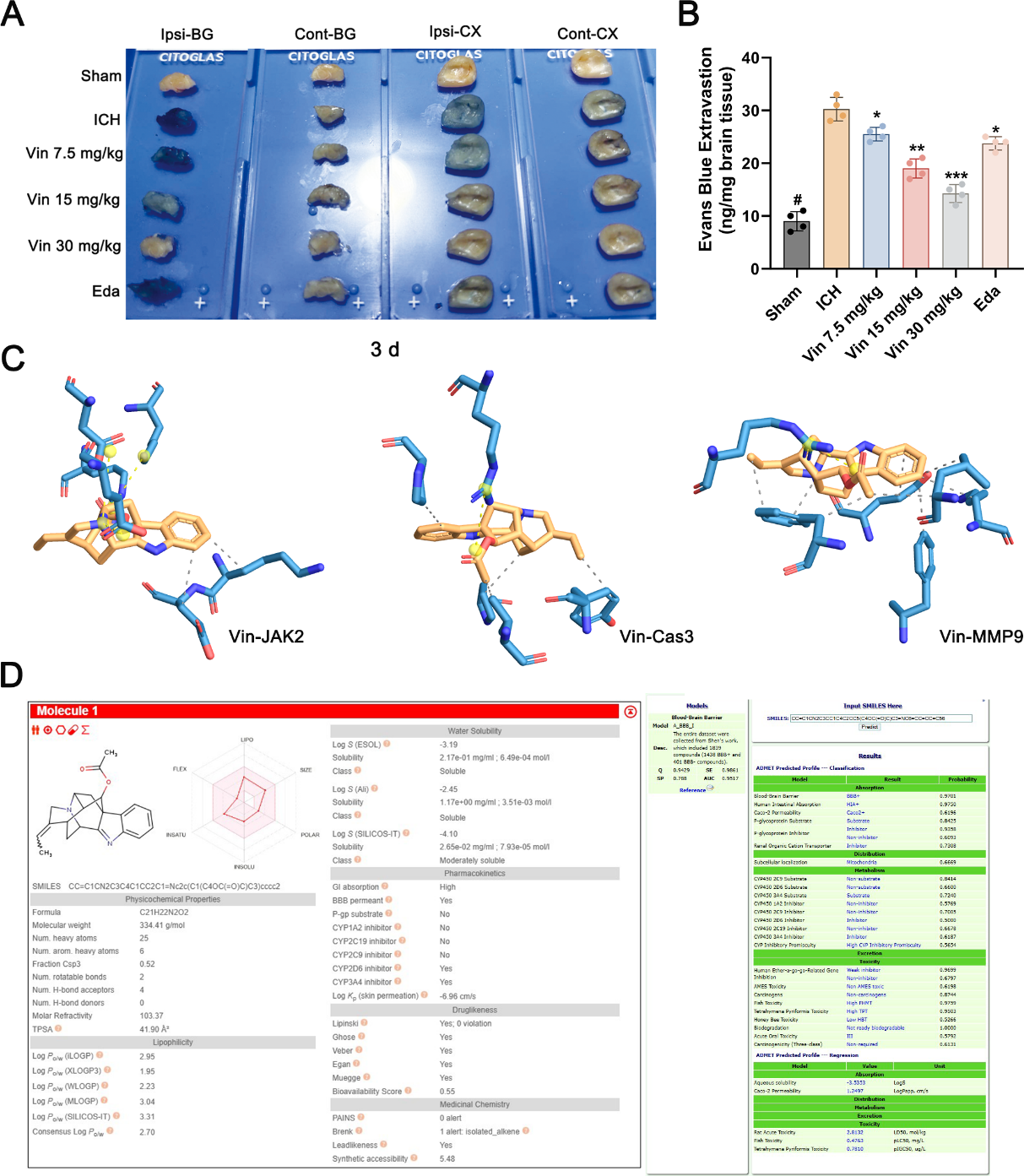
**

Figure S2. Evans blue and molecular docking related information. (A) Schematic diagram of Evans Blue dye staining of brain tissue. (B) Evans Blue-related statistical chart. (C) Schematic diagram of the docking mode of Vinorine with related proteins. (D) SwissADME website prediction of Vinorine-related information. All data were expressed as mean ± standard deviation (SD). Statistical significance was determined by two-way analysis of variance (ANOVA) and Tukey's multiple comparisons test, * p < 0.05, ** p < 0.01, *** p < 0.001 and ^#^ or **** p < 0.0001 VS ICH group, n ≥ 4. ns: not significant.


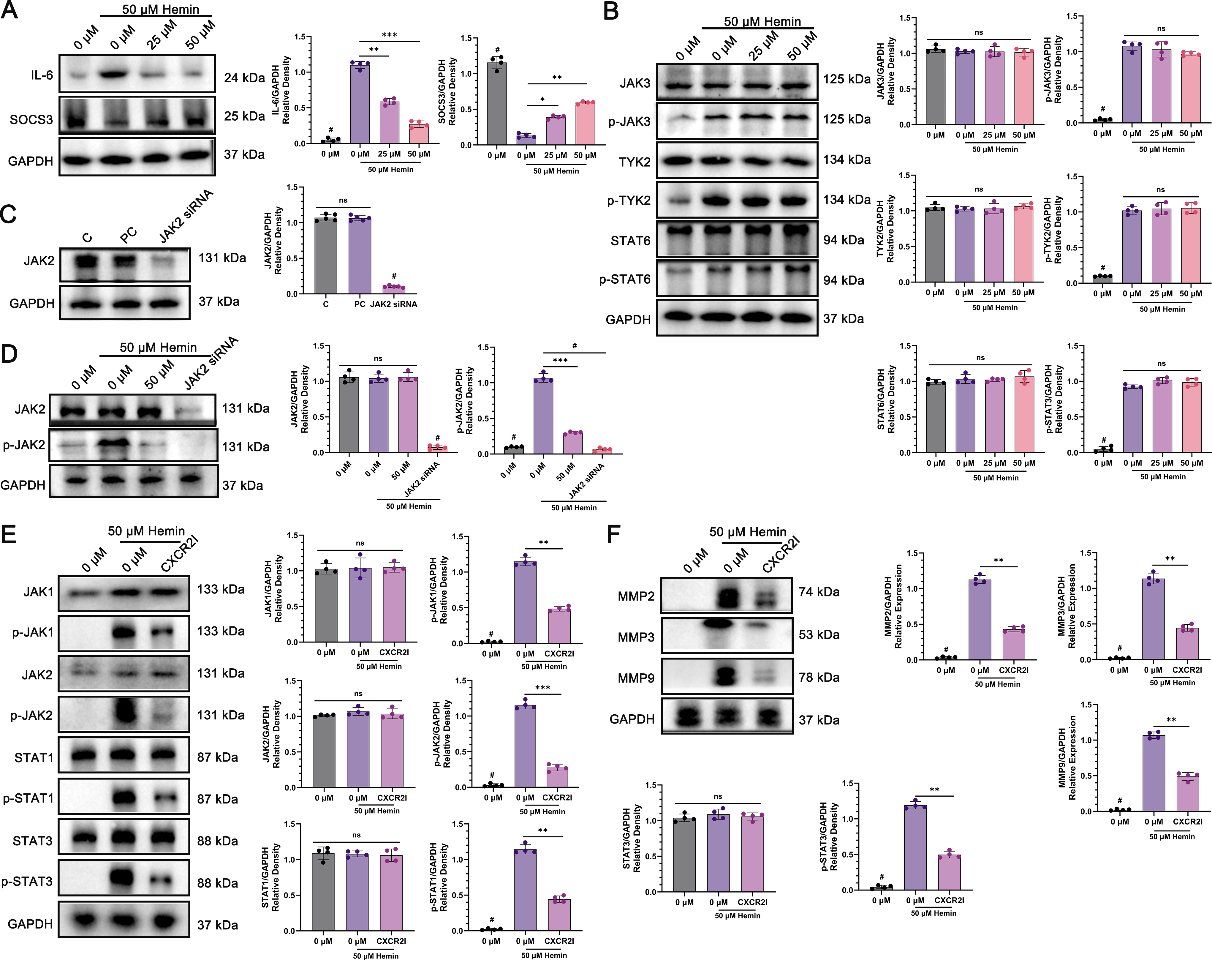


Figure S3. Effects of Vinorine on other proteins in the JAK-STAT pathway and downstream proteins. (A) The effect of Vinorine on IL-6 and SOCS3 proteins. (B) Effects of Vinorine on JAK3, TYK2, and STAT6 protein phosphorylation levels. (C) Validation of the efficacy of silent JAK2 protein plasmids. (D) Verification of the effects of Vinorine and JAK2 siRNA on JAK2 protein phosphorylation. (E-F) Verification of CXCR2-JAK-STAT axis activation in BV2 cells. All data were expressed as mean ± standard deviation (SD). Statistical significance was determined by two-way analysis of variance (ANOVA) and Tukey's multiple comparisons test, * p < 0.05, ** p < 0.01, *** p < 0.001 and ^#^ or **** p < 0.0001 VS 0 μM (50 μM Hemin pre-treated) group, n ≥ 4. ns: not significant.
